# A Bayesian framework for the analysis of systems biology models of the brain

**DOI:** 10.1371/journal.pcbi.1006631

**Published:** 2019-04-26

**Authors:** Joshua Russell-Buckland, Christopher P. Barnes, Ilias Tachtsidis

**Affiliations:** 1 Department of Medical Physics and Biomedical Engineering, University College London, London, United Kingdom; 2 Centre for Mathematics and Physics in the Life Sciences and Experimental Biology, University College London, London, United Kingdom; 3 Department of Cell and Developmental Biology, University College London, London, United Kingdom; Duke University, UNITED STATES

## Abstract

Systems biology models are used to understand complex biological and physiological systems. Interpretation of these models is an important part of developing this understanding. These models are often fit to experimental data in order to understand how the system has produced various phenomena or behaviour that are seen in the data. In this paper, we have outlined a framework that can be used to perform Bayesian analysis of complex systems biology models. In particular, we have focussed on analysing a systems biology of the brain using both simulated and measured data. By using a combination of sensitivity analysis and approximate Bayesian computation, we have shown that it is possible to obtain distributions of parameters that can better guard against misinterpretation of results, as compared to a maximum likelihood estimate based approach. This is done through analysis of simulated and experimental data. NIRS measurements were simulated using the same simulated systemic input data for the model in a ‘healthy’ and ‘impaired’ state. By analysing both of these datasets, we show that different parameter spaces can be distinguished and compared between different physiological states or conditions. Finally, we analyse experimental data using the new Bayesian framework and the previous maximum likelihood estimate approach, showing that the Bayesian approach provides a more complete understanding of the parameter space.

## Author summary

*Systems biology* models are mathematical representations of biological processes that reproduce the overall behaviour of a biological system. They are comprised by a number of parameters representing biological information. We use them to understand the behaviour of biological systems, such as the brain. We do this by fitting the model’s parameter to observed or simulated data; and by looking at how these values change during the fitting process we investigate the behaviour of our system. We are interested in understanding differences between a healthy and an injured brain. Here we outline a statistical framework that uses a *Bayesian* approach during the fitting process that can provide us with a distribution of parameters rather than single parameter number. We apply this method when simulating the physiological responses between a healthy and a vascular compromised brain to a drop in oxygenation. We then use experimental data that demonstrates the healthy brain response to an increase in arterial CO2 and fit our brain model predictions to the measurements. In both instances we show that our approach provides more information about the overlap between healthy and unhealthy brain states than a fitting process that provides a single value parameter estimate.

## Introduction

Systems biology models are used to understand complex biological and physiological systems comprised of large numbers of individual elements that give rise to emergent behaviours. These complex systems are dependent on both the properties of the whole network and on the individual elements [1]. This inherent complexity within the models can lead to difficulties in determining how best to interpret information obtained through their use.

At University College London, the family of BrainSignals models (and the BRAINCIRC model on which they are based) are used to understand the brain’s dynamics via a systems biology approach. They bring together a number of mathematical models relating to different aspects of blood circulation, oxygen transport and oxygen metabolism within the brain in order to develop a more complete model that can be used alongside experimental data to simulate physiological phenomena of the brain, such as autoregulation and neural activation. This allows us to understand how our measurements are linked to specific brain physiological and metabolic mechanisms.

All of the models were developed to reproduce broadband near-infrared spectroscopy (NIRS) measurements of brain tissue concentration changes of haemoglobin (oxygenation and haemodynamics) and cytochrome-*c*-oxidase (mitochondrial metabolism) and vary in their complexity and scope. The first model developed was the ‘BRAINCIRC’ model in 2005 [2], followed by the ‘BrainSignals’ model [3] in 2008. A number of additional versions were then developed from this, such as the ‘BrainPiglet’ model [4] which was developed to to simulate the physiological and metabolic processes of the piglet brain often used as the neonatal preclinical model. This was extended in BrainPiglet v2.0 to incorporate the effects of cell death during injury [5]. In 2015, Caldwell et al. modified and simplified the BrainSignals model to both reduce model complexity and improve model run time, producing the ‘BrainSignals Revisited’ model [6]. All of these models are run using the Brain/Circulation Model Developer environment (BCMD) and are defined in a simple text language. The data collected and analysed with the models primarily consists of broadband NIRS data, providing information about tissue oxygenation, through monitoring of oxy- and deoxy-haemoglobin levels, and cellular metabolism, through the concentration of cytochrome-c-oxidase. This data is then supplemented by systemic information such as blood pressure, arterial oxygen saturation and/or partial pressure of CO_2_.

One of the main uses of the models is to fit the model simulations to clinical and experimental data and investigate how model parameters are affected. In the case where data is collected from an injured or sick patient, these changes may illuminate what the underlying causes/mechanisms are behind the illness or injury is.

The models are currently fit using a maximum likelihood based method, with a single value obtained for each parameter. Sensitivity analysis performed on the models to determine which parameters are most important in influencing each model output for any particular dataset. These parameters are then optimised using the PSwarm method [7] to minimise a given error metric, such as the Euclidean distance, between the modelled and measured signals. Through this each output has a set of optimised parameter values. Parameter values were limited to the same ranges used in the sensitivity analysis [5].

This approach has a number of drawbacks. The models are mechanistic and, if fitted to single value parameter estimates, will produce the same output for the same input. Physiology and biology, however, is unlikely to operate in such a constrained manner. Additionally, this set of best-fit parameters for the model may not be representative of the full parameter space [8]. In an attempt to try to compensate for this potential drawback, Caldwell et al. [5] fit the BrainPiglet model multiple times for two different piglets and found that, whilst parameter values can vary within the same data, separate parameter spaces for each piglet did seem to exist based on the brain physiological status of the piglet following a hypoxic-ischaemic insult.

One of the key ways in which these models are used to extract information from data is through the use of parameter estimation and fitting. However, this step remains a difficult mathematical and computational problem, potentially originating in the lack of identifiability [9]. In addition, there has been discussion of ‘universal sloppiness’ within dynamic systems biology models. Gutenkunst et al. [10] proposed that *sloppiness*, where the parameters of a dynamic model can vary by orders of magnitude without affecting model output, is a universal property of systems biology models. Due to this sloppiness, it may not be possible to make parameter estimations that can be used to make inferences about the system [10, 11]. Chis et al. have stated however that sloppiness is not equivalent to a lack of identifiability and that a sloppy model can still be identifiable [12]. Apgar et al. note that experimental design can be used to constrain a sloppy parameter space by choosing a set of complementary experiments [13].

The use of a Bayesian methodology, by avoiding point estimates, can allow the full uncertainty of the problem to be captured [8]. In fact, the use of an Approximate Bayesian Computation (ABC) approach, discussed below, is particularly well suited to these kinds of problems [14]. There are many examples of Bayesian methods being used to analyse bioinformatics data and systems biology models [15], including in sequence analysis [16], gene microarray data [17] and in models of genetic oscillators [18] and DNA network dynamics [19]. There are a number of models that take a systems biology approach towards understanding physiology, particularly oxygen transport and blood flow, including the previously mentioned BrainSignals [2, 3, 5] and BrainPiglet [4, 5] models, the Aubert-Costalat model [20], and work by Fantini [21–24] and Orlowski and Payne [25, 26] where Bayesian parameter estimation could also be applied but has yet to be.

Bayesian inference utilises Bayes’ rule,

$$\begin{array}{c} p\left(\theta |y\right)=\frac{p(\theta ,y)}{p(y)}=\frac{p(\theta )p(y|\theta )}{p(y)}, \end{array}$$

where *p*(*y*) = ∫_*θ*_ *p*(*θ*)*p*(*y*|*θ*)*dθ* is the marginal probability of *y* and *p*(*y*|*θ*) is the *likelihood*. Typically, *p*(*y*) is not known and the likelihood will not be known explicitly or may require marginalising over some values of *θ*. This often leaves the solution analytically intractable. Instead we can try solve for *p*(*θ*|*y*) using a Monte Carlo or Markov Chain Monte Carlo (MCMC) approach.

Where a likelihood function can be defined there are a number of these methods that can be used to infer a posterior distribution, *p*(*θ*|*y*). The simplest is the Gibbs Sampler [27], which in its most basic form is a special case of the Metropolis-Hastings algorithm [28]. Although the BrainSignals models are deterministic, the model noise is a combination of process noise and experimental error which is expected to depend on the state in a non-trivial manner. This makes formulating an analytical expression for the likelihood difficult. In this case where a likelihood expression is unobtainable a likelihood-free approach using ABC is required instead. There are a number of different methods available with the simplest being the ABC rejection algorithm (ABC REJ) approach. This has the additional benefit of allowing us to consider different summary statistics that would not be valid in a likelihood-based approach. It may be that these summary statistics allow us to optimise for specific behaviours that have physiological relevance.

The aim of this paper is to introduce the new *BayesCMD* modelling platform that can be used in systems biology models of physiology such as the BrainSignals models, but that can be replicated beyond these. For this work, we have chosen to use ABC REJ as whilst it is less efficient than the other methods mentioned here, the simplicity with which it can be implemented is a significant factor. The models and modelling environment used are already complex and so this initial work focuses on the use of the simplest method as proof of utility. We will demonstrate the effectiveness of this approach by using it to analyse two simulated datasets chosen to represent healthy and impaired brain states, before then using it on experimental data from a healthy subject undergoing a hypoxia challenge. We will show that the Bayesian approach allows us to extract more information from our data than the previous maximum likelihood approach, with a more complete picture of the parameter space being obtained.

## Materials and methods

**Fig 1:**
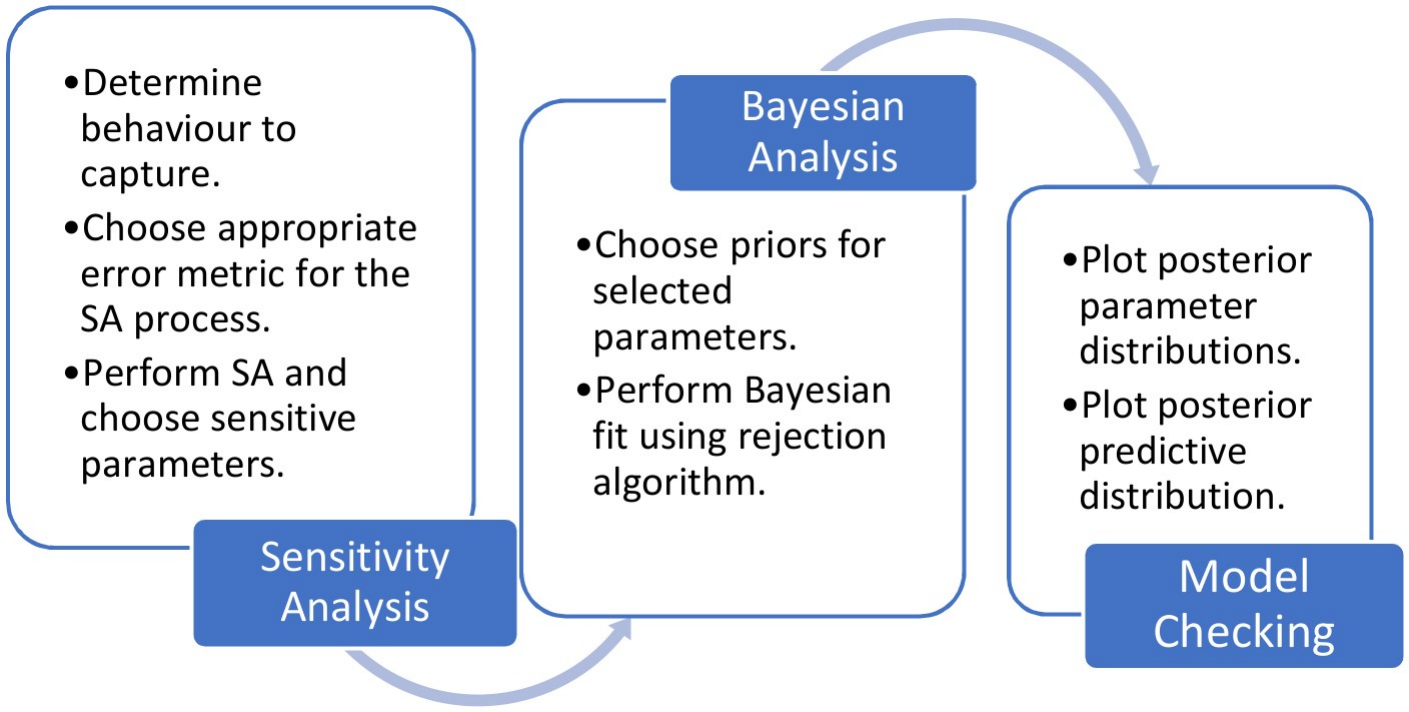
Generalised analysis process. A simplified representation of the Bayesian analysis process.

Fig 1 shows a generalised outline of the final Bayesian analysis process. It can be split into three main sections: sensitivity analysis, Bayesian analysis and model checking. However, before applying the process, data must be generated or collected and an appropriate model chosen.

### Choice of model

Whilst a brief overview of the history of the BrainSignals models was given in the introduction, in this section we provide more information about the specifics of the different models. Table 1 compares the number of reactions, equations, relations, reactions, variables and parameters in three different models. The BRAINCIRC model from 2005 built on an earlier circulatory model by Ursino and Lodi [29] and combined models for the biophysics of the circulatory system, the brain metabolic biochemistry and the function of vascular smooth muscle. The BrainSignals model which succeeded it simplified the ‘BRAINCIRC’ model and added a submodel of mitochondrial metabolism. As previously mentioned, in order to better simulate the physiological and metabolic processes of the piglet brain, which is often used as the neonatal preclinical model, the ‘BrainPiglet’ model [4] was developed from the BrainSignals model. It involved modifying the default values for 11 of the 107 parameters used and was extended to include simulated measurements for magnetic resonance spectroscopy values that included brain tissue lactate and ATP production, measurements of which are available in piglet studies. Its extension, BrainPiglet v2, incorporated the effects of cell death during injury in order to to investigate why two piglets showed different recoveries following hypoxia-ischaemia, finding that the differences could be explained by including cell death within the model [5].

**Table 1:** Comparison of the number of reactions, equations, relations, reactions, variables and parameters in the BRAINCIRC, BrainSignals Revisited and BrainPiglet v2.0 models.

	BRAINCIRC	BrainSignals Revisited	BrainPiglet v2.0
Reactions	81	5	17
Differential Equations	5	9	21
Algebraic Relations	72	3	3
Variables	168	40	128
Parameters	697	139	227

The ‘BrainSignals Revisited’ model was produced by making various simplifications to the BrainSignals model by identifying various functions that could be replaced by linear approximators without reducing model applicability. This reduced complexity and decreased the time taken to run a simulation, whilst being able to reproduce the same results and behaviour of the original model. This reduced model of the adult brain was later extended to simulate extracerebral haemodynamics to investigate confounding factors with brain near-infrared spectroscopy measurements, the ‘BSX’ model [30].

**Fig 2:**
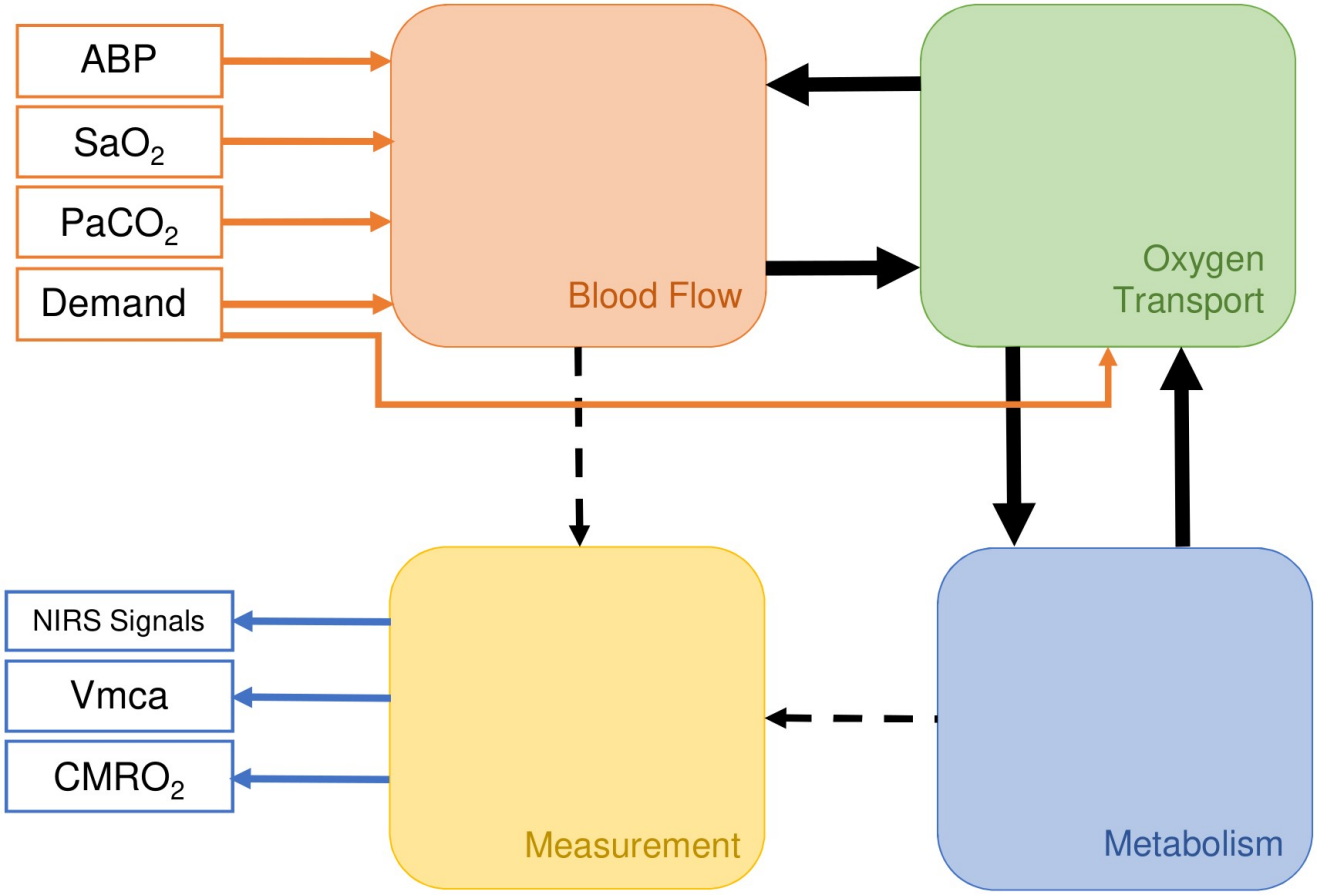
Simplified structure of a typical BrainSignals model. A typical BrainSignals model can be split into four compartments or submodels. The *blood flow* submodel represents blood flow from arteries to veins via the capillary bed and the *oxygen transport* submodel estimates diffusion of dissolved O_2_ from the capillary blood to the brain tissue. Delivered oxygen is then utilised by the *metabolism* submodel. Finally, the *measurement* submodel translates the internal states of the blood flow and metabolism submodels into observable outputs. Model inputs are shown in red and consist of arterial blood pressure (ABP), arterial oxygen saturation (SaO2), partial pressure of CO_2_ (PaCO2) and a parameter specifying relative demand, whilst measurable outputs are shown in blue, including NIRS signals as well as middle cerebral artery velocity (Vmca) and cerebral metabolic rate of oxygen (CMRO_2_).

The models are driven with input signals, such as the blood pressure and/or oxygen saturation, and simulate brain tissue measurments of oxygenation, blood volume and metabolism, as well as the middle cerebral artery velocity (Vmca) and the cerebral metabolic rate of oxygen (CMRO_2_). The model can be split into roughly 3 compartments—blood flow, oxygen transport and metabolism—with boundaries chosen to minimise interdependence. Fig 2 outlines this in more detail.

All of these models are solved using the BCMD framework and are written in a simple text format that can be translated to executable C code and solved using the RADAU5 solver [31]. The models take a standard differential-algebraic equation representation, of the form:

$$\begin{array}{c} M\frac{dy}{dt}=f\left(y,\theta ,t\right) \end{array}$$ (1)

where **y** is a vector of variables of interest, **M** is a constant, possibly-singular, mass matrix specifying relations among the differential terms, and **f** is some vector-valued function, possibly having additional parameters ***θ***. If a row of **M** is zero, the corresponding equation in **f** is algebraic rather than differential.

In this work we have chosen to use the refactored BrainSignals model [6], with a minor modification to include the haemoglobin difference (ΔHbO_2_ − ΔHHb = ΔHbD) as a model output alongside the normal outputs of oxyhaemoglobin (ΔHbO_2_), deoxyhaemoglobin (ΔHHb), total haemoglobin (ΔHbO_2_ + ΔHHb = ΔHbT), tissue oxygenation index (TOI), and cytochrome-*c*-oxidase (ΔCCO). Both ΔHbD and ΔHbT are included in the experimental dataset due to them being good indicators of brain oxygenation changes and brain blood volume changes respectively, with both being easily measured using broadband NIRS. All NIRS outputs, except TOI, are measured as changes relative to an initial value and therefore both data and model outputs are normalised to an initial value of 0.

### Data

Three datasets were used to test the new Bayesian model analysis process. Firstly, ‘healthy’ data was simulated using the BrainSignals model with the default parameter settings, as per [2, 3]. Next, the same inputs were used but with the model modified to represent an ‘impaired’ brain. To do this, a single parameter was changed to reflect a potential pathology or injury, to generate an ‘impaired’ simulated dataset. Finally, we used experimental data from a healthy adult undergoing a hypoxia challenge.

#### Simulated data

**Fig 3:**
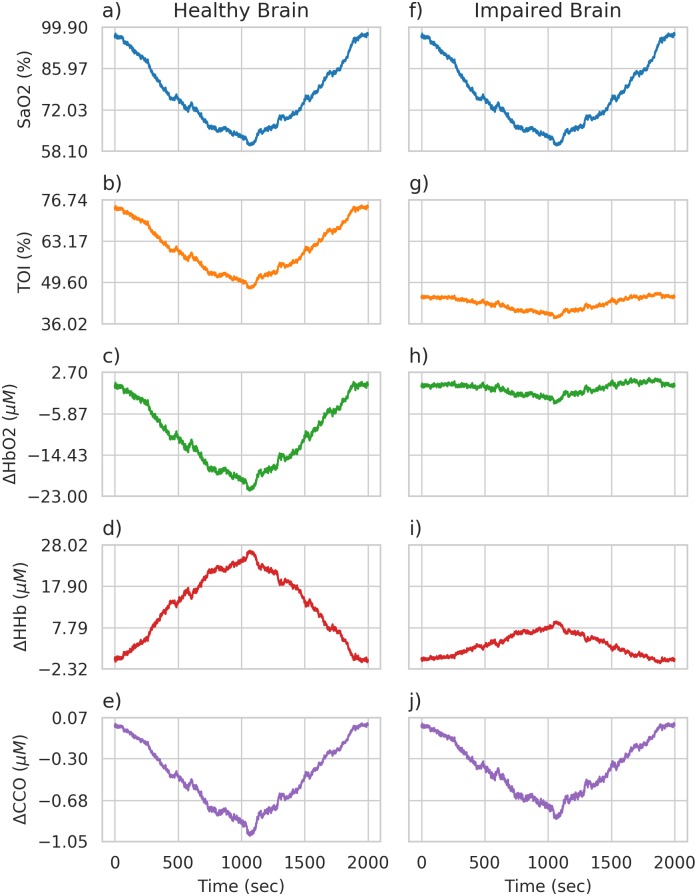
Healthy and impaired brain simulations. Figures a)-e) show simulations of a healthy brain’s response to hypoxia, whilst f)-j) show the impaired brain’s response. The input variable of arterial oxygen saturation is shown in blue and is the same for both simulations, whilst the outputs of TOI, ΔHbO_2_, ΔHHb and ΔCCO clearly differ between the two brain states.

Partial pressure of CO_2_ (PaCO_2_) and arterial blood pressure (ABP) were kept at their baseline values of 40 mmHg and 100 mmHg respectively, whilst arterial oxygen saturation (SaO_2_) was varied to simulate hypoxia through a decrease in arterial oxygen saturation from 97% to 65%. Initially, all model parameters were kept at their default values in order to simulate a healthy brain’s response to this challenge. Fig 3 shows the arterial saturation data and the model response across all considered model outputs.

**Fig 4:**
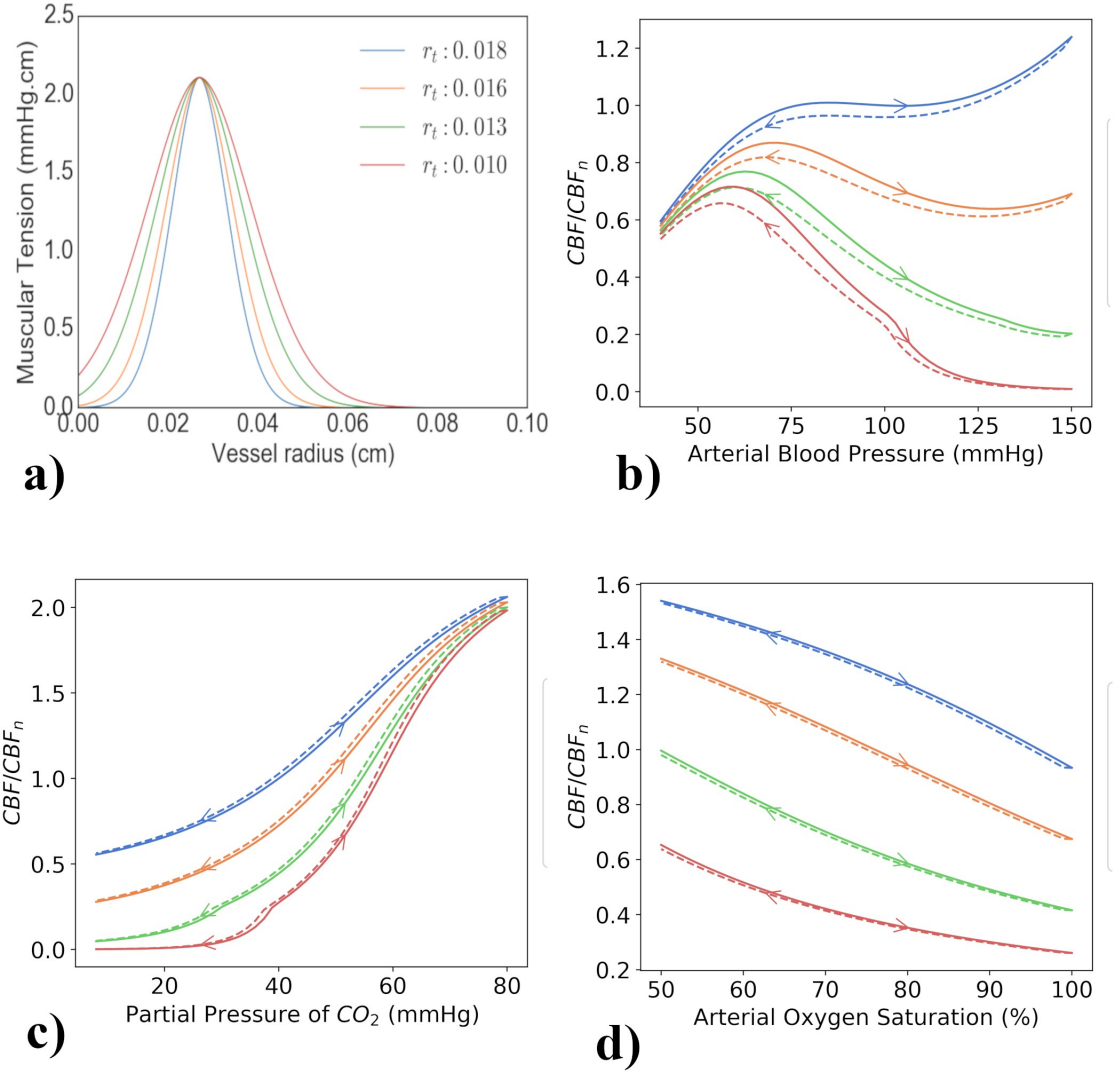
Fig 4a shows the effect of different *r_t_* values on the shape of the muscular tension curve for a range of vessel radii. It can be seen that reducing *r_t_* widens the curve, leading to increased muscular tension for the same vessel radius. Figures 4b, 4c and 4d show the effect of both increasing and decreasing model inputs on cerebral blood flow for different values of *r_t_*. Cerebral blood flow (CBF) is given as a proportion of the normal CBF (40 ml 100g^−1^ min^−1^). Changing *r_t_* has a significant effect on the brain’s ability to autoregulate within the model. Fig 4b shows that higher blood pressures causes a decrease in cerebral blood flow for lower *r_t_*, as opposed to an increase at the normal value of *r_t_* = 0.018 cm. Fig 4c shows that for lower *r_t_* values, CBF decreases quicker as PaCO_2_ is decreased. Fig 4d shows that across all considered oxygen saturations, lower *r_t_* gives a lower CBF.

After simulating the healthy brain response and determining its posterior parameter distribution, the model was altered to include a pathological or impaired brain state. Fig 3f)–3j) shows the model response across all considered model outputs for this impaired brain state. The response of the model outputs to the same change in arterial saturation is much smaller than in the healthy simulation, with the TOI having a lower baseline value of around 45% as compared to around 75%. This was done by changing a single parameter to be outside of the healthy parameter space. r_t, which affects the shape of the muscular tension relationship, was found to be sensitive in both the sensitivity analysis process (see Simulated data in the sensitivity analysis results) and the Bayesian analysis. This is clearly seen in its comparatively narrow marginal posterior for the healthy data. Stifening of blood vessels in the brain has also been noted as a potentially important factor in a number of different pathologies, including Alzheimers [32], and in autoregulation, as seen in Fig 4.

The muscular tension relationship is defined as

$$\begin{array}{c} T_{m}=T_{max}\text{exp}(-{\left|\frac{r-r_{m}}{r_{t}-r_{m}}\right|}^{n_{m}}), \end{array}$$ (2)

where *T*_*m*_ is the muscular tension within the vessel wall and has a bell-shaped dependence on the vessel radius, taking value *T*_*max*_ at some optimum radius *r*_*m*_. *r*_*t*_ and *n*_*m*_ are parameters determining the shape of the curve. Fig 4a illustrates the effect of changing *r*_*t*_ on the shape of the curve and shows that decreasing *r*_*t*_ leads to increased muscular tension for the same vessel radius due to a widening of the bell-shaped curve. This can be seen to represent a stifening of vessels within the brain.

Changing *r*_*t*_ has a significant effect on the brain’s ability to autoregulate within the model as seen in Fig 4b, 4c and 4d. Fig 4b shows that higher blood pressure causes a decrease in cerebral blood flow (CBF) for lower *r*_*t*_ values, as opposed to an increase at the normal value of *r*_*t*_ = 0.018 cm. Fig 4c shows that CBF is lower and decreases quicker for lower *r*_*t*_ values as PaCO_2_ is decreased and Fig 4d shows that across all considered oxygen saturations, lower *r*_*t*_ gives a lower CBF.

Whilst we would expect impairment of a real biological system to stem from multiple parameter changes the intention here was to make the simplest modification possible whilst still representing a potentially real physiological change in order test the method under the simplest conditions. Additionally, it should be noted that a single parameter change will have effects on various physiological variables. As outlined below, we also apply the method to experimental data which is inherently more complex than this simple example and where we expect multiple parameters to differ from baseline.

#### Experimental data

**Fig 5:**
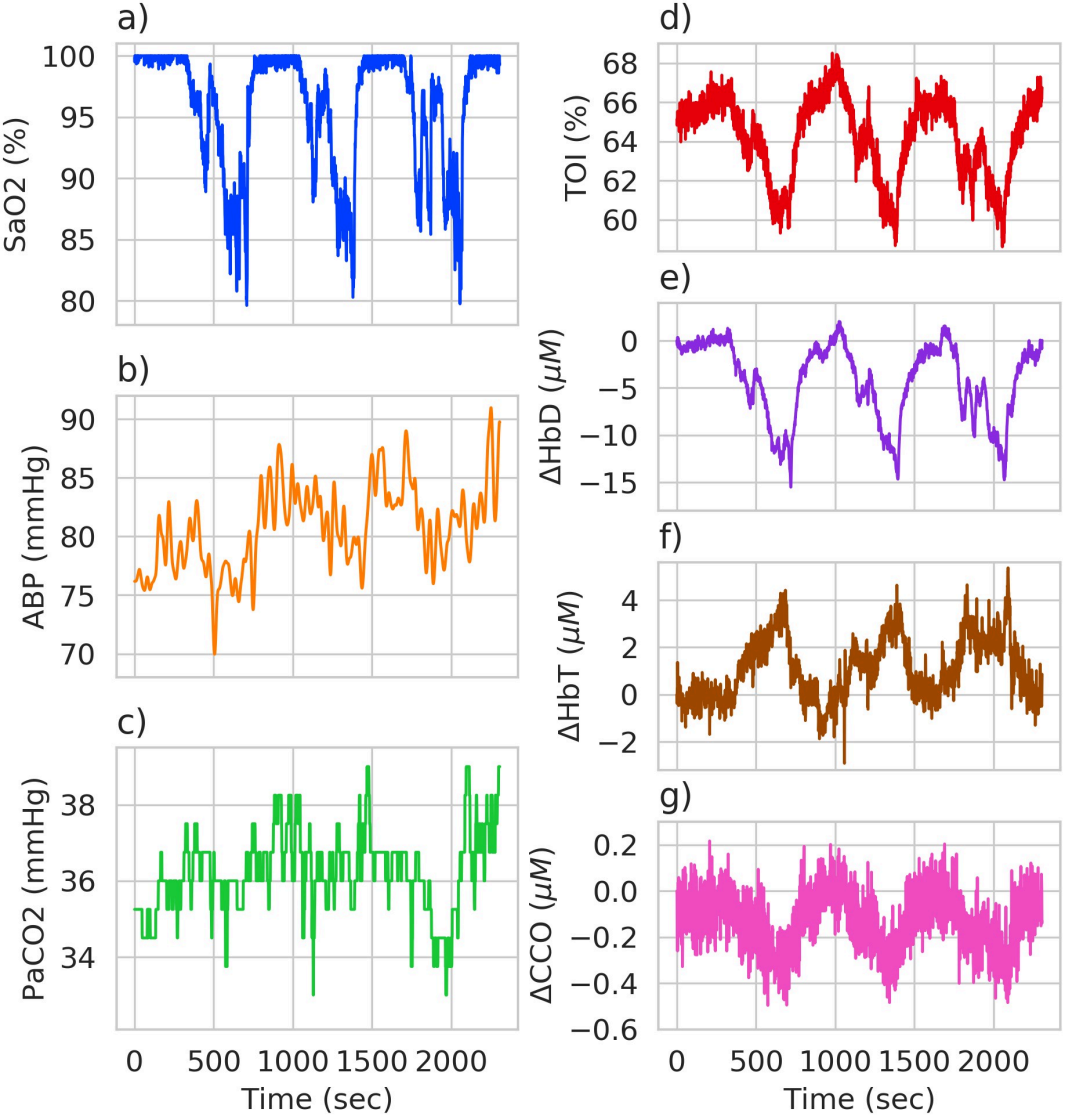
Experimental hypoxia data. Data collected from a healthy adult during a hypoxia challenge. Systemic data used as model inputs are shown in figures a), b) and c), with broadband NIRS measurements shown in figures d), e), f) and g).

Experimental data will inherently contain more uncertainty for parameter fitting than data generated by the model itself. This makes it important to test the Bayesian analysis process on experimental data as well as that simulated from the model. The data used was originally collected by Tisdall et al. [33] and is shown in Fig 5. Healthy adult humans had their arterial oxygen saturation reduced from baseline to 80%, whilst minimising changes in end tidal carbon dioxide tension (EtCO_2_).

The dataset contains three model inputs: arterial oxygen saturation, end tidal CO_2_ and arterial blood pressure, with EtCO_2_ converted to partial pressure of CO_2_. Blood pressure data was filtered using a low pass 5th order Butterworth filter, with a cut off of 0.05 Hz, to remove noise. The heavily quantised nature of the partial pressure of CO_2_ data is not an issue here as the model contains first order filters to smooth input signals over a given time period.

In terms of model outputs, only NIRS signals were used: ΔHbD, ΔHbT, ΔCCO and TOI. All data was resampled to 1 Hz.

### Sensitivity analysis

When fitting a model as complex as BrainSignals, it is important to reduce the number of parameters that are required to be fit. We expect that not all parameters will have a significant impact on the model output for given set of input data. Instead, we can attempt to reduce the number of considered parameters through sensitivity analysis. We used the Morris method [34, 35], which is known to work well with a large number of parameters. The method requires the time series to be reduced to a single number and identifies the parameters that have produce the most variance in this summary value. Previously, we have used the Euclidean distance over the whole time series as our summary value but this has a number of significant drawbacks.

**Fig 6:**
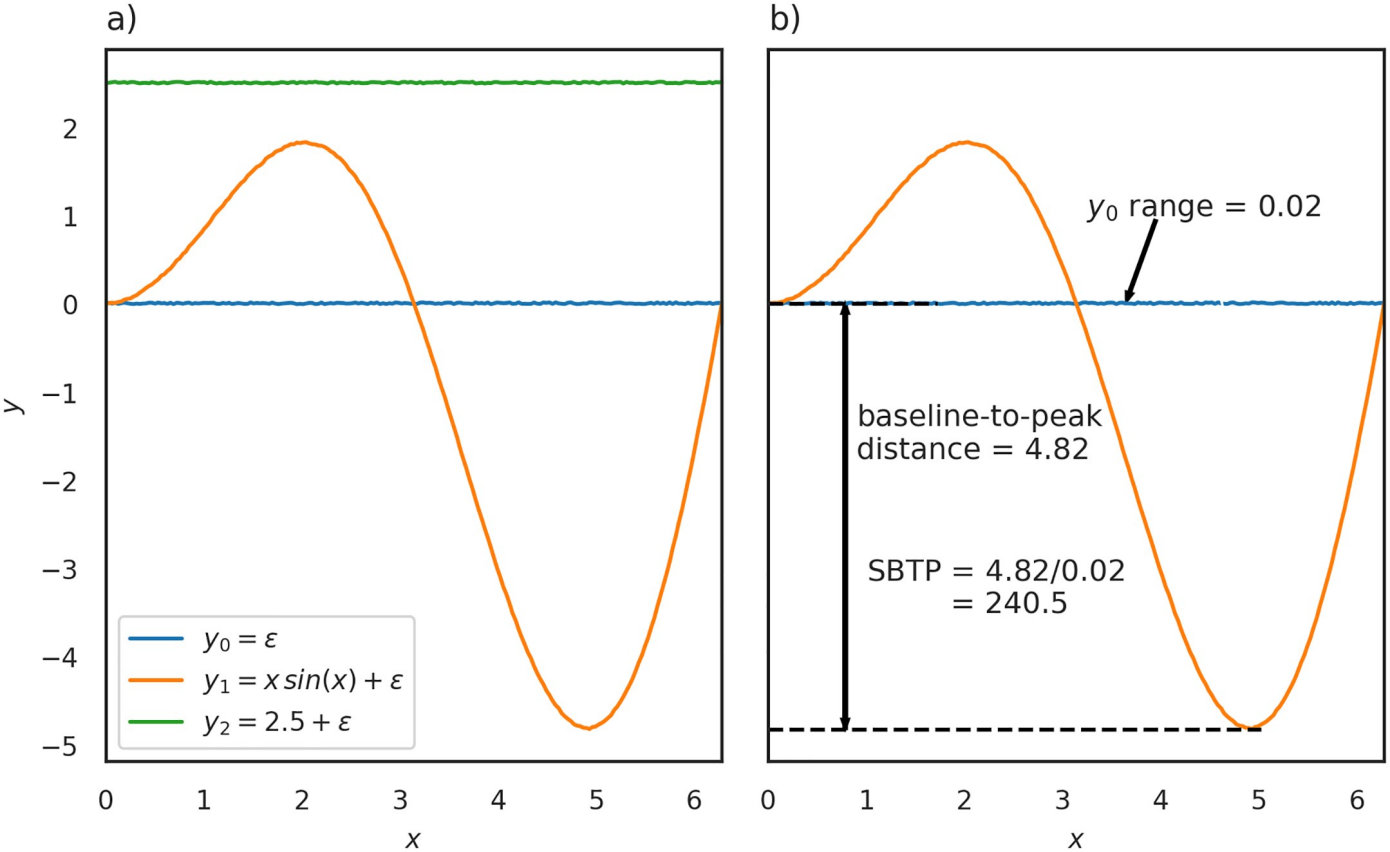
Fig 6a shows data generated from the same test function *y_i_* = *a* *x* sin(*x*) + *b* + *ϵ*, where *a*, *b* are both model parameters and *ϵ* is random Gaussian noise. *x* was varied from 0 to 2*π*, producing data *y*_0_, *y*_1_ and *y*_2_ for the parameter sets Θ_0_: *a* = 0, *b* = 0, Θ_1_: *a* = 1, *b* = 0 and Θ_2_: *a* = 0, *b* = 2.5 respectively. Despite both *y*_1_ and *y*_2_ being qualitatively very different they are very similar when summarised using only the Euclidean distance, with *y*_1_ having a Euclidean distance *ε*_euc,1_ = 35.58 and *y*_2_ having a Euclidean distance *ε*_euc,2_ = 35.44. If we instead look at the scaled baseline-to-peak (SBTP) distance we find that *y*_1_ has a SBTP distance *SBTP*(*y*_1_) = 240.5 and *y*_2_ has a SBTP distance *SBTP*(*y*_2_) = 0.27, giving *ε*_SBTP,1_ = 240.2 and *ε*_SBTP,2_ = 0.11. Fig 6b illustrates how the scaled baseline-to-peak distance is defined using *x* sin(*x*) + *ϵ* as the example signal. The baseline-to-peak distance is the absolute distance from the baseline to max ({|*y_max_*|, |*y*_*min*_|}). This is then divided by the range of the ‘default’ data, *y*_0_, to get the distance as a proportion of the total change seen within the data. In this example, baseline-to-peak distance is 4.82 and the range is 0.02, giving the previously mentioned SBTP distance of 240.5.

If the summary measure is the distance across the whole time series, we’re failing to capture specific changes that we know to be physiologically important. In the case of our hypoxia simulation, for example, we want to select parameters that are important in controlling the overall change from baseline. Taking the Euclidean distance over the time series as a whole however does not prioritise this behaviour. Fig 6a shows three sets of data generated from the same toy model function

$$\begin{array}{c} y_{i}=a\;x\;\text{sin}\left(x\right)+b+\epsilon , \end{array}$$ (3)

where *a*, *b* are both model parameters and *ϵ* is random Gaussian noise.

Assume that without modification, our model produces data *y*_0_, with the default parameters Θ_0_: *a* = 0, *b* = 0, and that the behaviour we want to reproduce is sinusoidal but, for some reason, we don’t know which parameter is most important in producing this specific behaviour. We decide to undertake sensitivity analysis, using a distance measure of some kind as our summary statistic in order to identify the parameter most important in producing sinusoidal behaviour. If when altering a parameter that distance measure increases, then the behaviour summarised by that distance is sensitive to changes in that parameter. In this case, to produce sinusoidal behaviour, we would want parameter *a* to be identified as important rather than parameter *b*.

To generate our data *x* was varied from 0 to 2*π*, producing datasets *y*_1_ and *y*_2_ for the parameter sets Θ_1_: *a* = 1, *b* = 0, where only *a* is changed from baseline, and Θ_2_: *a* = 0, *b* = 0.707, where only *b* is changed from baseline, respectively. *y*_0_ and parameter set Θ_0_ provide our baseline data. This is seen in Fig 6a. It is clear from the figure that the two outputs *y*_1_ and *y*_2_ show very different behaviour, the behaviour we want to optimise for is seen in *y*_1_.

Despite both *y*_1_ and *y*_2_ being qualitatively very different they are very similar when summarised using only the Euclidean distance, with *y*_1_ having a Euclidean distance *ε*_euc,1_ = 10.01 and *y*_2_ having a Euclidean distance *ε*_euc,2_ = 10.03. This means that we would fail to clearly identify parameter *a* as being important than parameter *b* in producing sinusoidal behaviour.

Instead we can define a new summary measure, which we will call the “scaled baseline-to-peak” (SBTP) distance. We know that we want to find the parameter that determines how sinusoidal our model is. One way to emphasise this behaviour is to find the distance from our baseline to the maximum or minimum (whichever has the largest absolute value) of our data, as illustrated in Fig 6b. We then scale this by the range of our ‘default’ signal, *y*_0_, to normalise it and avoid issues comparing data of different magnitudes. This gives us

$$\begin{array}{c} SBTP\left(y_{i}\right)=\frac{\text{max}(\{|\text{max}\left(y_{i}\right)-y_{i}\left(t=0\right)|,|\text{min}\left(y_{i}\right)-y_{i}\left(t=0\right)|\})}{\text{max}\left(y_{0}\right)-\min\left(y_{0}\right)}) \end{array}$$ (4)

We then find the Euclidean distance between the SBTP value for our ‘default’ data, *SBTP*(*y*_0_), and *SBTP*(*y*_1_) and *SBTP*(*y*_2_)

$$\begin{array}{c} \varepsilon_{SBTP,\;i}=\sqrt{(SBTP\left(y_{0}\right)-SBTP\left(y_{i}\right){)}^{2}}, \end{array}$$ (5)

where here *i* ∈ {1, 2}.

If we use *ε*_*SBTP*_ as our summary measure, we find that *y*_1_ has a distance *ε*_SBTP,1_ = 240.2 and *y*_2_ has a distance *ε*_SBTP,2_ = 0.11. This would mean that parameter *a* could be clearly identified as being more important in producing sinusoidal behaviour than parameter *b*.

We scale our baseline-to-peak distance because a number of model outputs significantly vary over different scales. For example, cerebral oxygenation can be measured through TOI which is a percentage and, as seen in Fig 3 can vary over 10-20%. Cytochrome-*c*-oxidase however, varies over a much smaller range, with a change of less than 1μM being typical. Failing to account for these different scales will lead to parameters that affect larger magnitude outputs being identified as more sensitive than those that affect smaller magnitude outputs, even if the relative change is significant.

For example, if changing a parameter *θ*_1_ causes the CCO change seen in Fig 3e) to double to a minimum of -2μM, whilst a change in a parameter *θ*_2_ causes TOI to decrease to 55%, without scaling the model seems more sensitive to *θ*_2_ because the magnitude of the change is much more, even though the relative change is smaller. If we consider this change proportional to the range of our data however, we account for its relative size.

It should also be noted that this choice of metric is specific to the behaviour being optimised for. For example, in the case of a signal that is non-oscillatory, a different summary method would be required based around the behaviour to be replicated within that particular signal. We also acknowledge that there are a variety of different methods for identifying a sinusoidal signal from a linear signal and that our choice of metric here is one of many. We have chosen it as in the case of our hypercapnia data, we expect to see our signal to change from baseline to maxima or minima, depending on the signal, before then returning to baseline. The SBTP distance emphasises this behaviour in a single number whilst also being easily comparable to previous work where the Euclidean distance was used.

We used the Morris elementary effect method [34] variant devised by Saltelli et al. [36]. This provides us with two notable statistics: the mean of the absolute values of the changes, μ_*_, and their standard deviation, *σ*. The larger the value of μ_*_, the more influential parameter is on the output, whilst the larger the standard deviation, the more non-linear the influence of the parameter is. The top ten most sensitive parameters, as per μ_*_ were chosen to fit the model. *σ* was not used to determine which parameters to fit as, whilst knowing the non-linearity of a parameter is useful, in previous work [5, 6] we have opted to use simply μ_*_ as this gives a good summary of the sensitivity of a single parameter and feel it is pertinent to continue to do so here. The parameter range considered for sensitivity is the default value ±50%. Sensitivities are calculated for each output as well as across all outputs jointly. This joint sensitivity is calculated by summing the SBTP value for each output and then determining variability in this total.

### Approximate Bayesian computation

After selecting the most important parameters, the model was fit using the rejection algorithm [37]. This is defined, as per [38], as:

1. Sample a candidate parameter vector θ* from the proposal distribution *p*(*θ*).
2. Simulate a dataset *y*^*rep*^ from the model described by a conditional probability distribution *p*(*y*|θ*).
3. Compare the simulated dataset, *y*^*rep*^, to the experimental dataset, *y*, using a distance function, *d*, and tolerance, *ϵ*. If *d*(*y*, *y*^*rep*^) ≤ *ϵ*, accept θ*. The tolerance *ϵ* ≥ 0 is the desired level of agreement between *y* and *y*^*rep*^.

The output of the ABC algorithm used will be a sample from the distribution *p*(*θ*|*d*(*y*, *y*^*rep*^) ≤ *ϵ*). If *ϵ* is sufficiently small, then *p*(*θ*|*d*(*y*, *y*^*rep*^) ≤ *ϵ*) will be a good approximation for the posterior *p*(*θ*|*y*).

The choice of *d*(⋅, ⋅) is important, just as with the sensitivity analysis. Previously the Euclidean distance has been used to fit the model but, as in the case of the sensitivity analysis, this fails to account for outputs that vary over different magnitudes. Instead, we have chosen to include a number of other distance metrics including the root-mean-square error (RMSE) and the normalised root-mean-square error (NRMSE). These are defined as

$$\begin{array}{c} RMSE=\sqrt{\frac{\sum_{t=1}^{T}{\left(x_{1,t}-x_{2,t}\right)}^{2}}{T}} \end{array}$$ (6)

$$\begin{array}{c} NRMSE=\frac{RMSE(x_{1},x_{2})}{x_{1,max}-x_{1,min}} \end{array}$$ (7)

where **x**_1_ and **x**_2_ are the two time series being compared, running over *t* = 1 to *t* = *T*, with *T* being the total number of time points.

By dividing the RMSE by the range of the data, the errors for time series that vary over different magnitudes are comparable. Without doing this, parameters that mainly affect outputs that vary over larger magnitudes are preferentially optimised. Normalisation prevents overfitting of one output at the expense of others, providing a more reliable joint posterior distribution after fitting.

After an initial exploratory fitting of the different datasets, it was found that setting an absolute tolerance value was not a suitable selection criteria. This was due to massively differing distance values between datasets, with all parameter combinations in the simulated healthy dataset producing NRMSE values smaller than almost all parameter combinations on the impaired dataset.

In general, the number of accepted samples that gives an adequate approximation of the posterior distribution is problem dependent; dispersed posterior distributions will ultimately require more samples. Poor estimation of the posterior can in most cases result in a wide posterior predictive distribution which appears to give a poor quality fit because outlier posterior samples cause biases. To address this issue in a pragmatic way, a fixed acceptance rate of 0.01% was set. This meant the 0.01% parameter combinations with the lowest *d*(*y*, *y*^*rep*^) were used as the posterior. The posterior was visualised through kernel density estimation on a pairplot using the Seaborn plotting package [39]. The posterior predictive density is then generated by sampling directly from the posterior 25 times and the model simulated for each sample. The results are aggregated and plotted, with the median and 95% credible interval marked on the plot.

The model was run in batches of 10,000,000 and the parameter combinations within the acceptance rate were used as a posterior. This batch size was chosen as a compromise between sufficient sampling of the parameter space and the computational time required to run the batch. The quality of the fit obtained from this posterior determined if the model had been run a sufficient number of times to sample the posterior adequately. If the posterior predictive distribution failed to capture the behaviour seen in the “true” data, then the process was repeated until a more adequate fit was obtained.

## Results

### Sensitivity analysis

#### Simulated data

**Fig 7:**
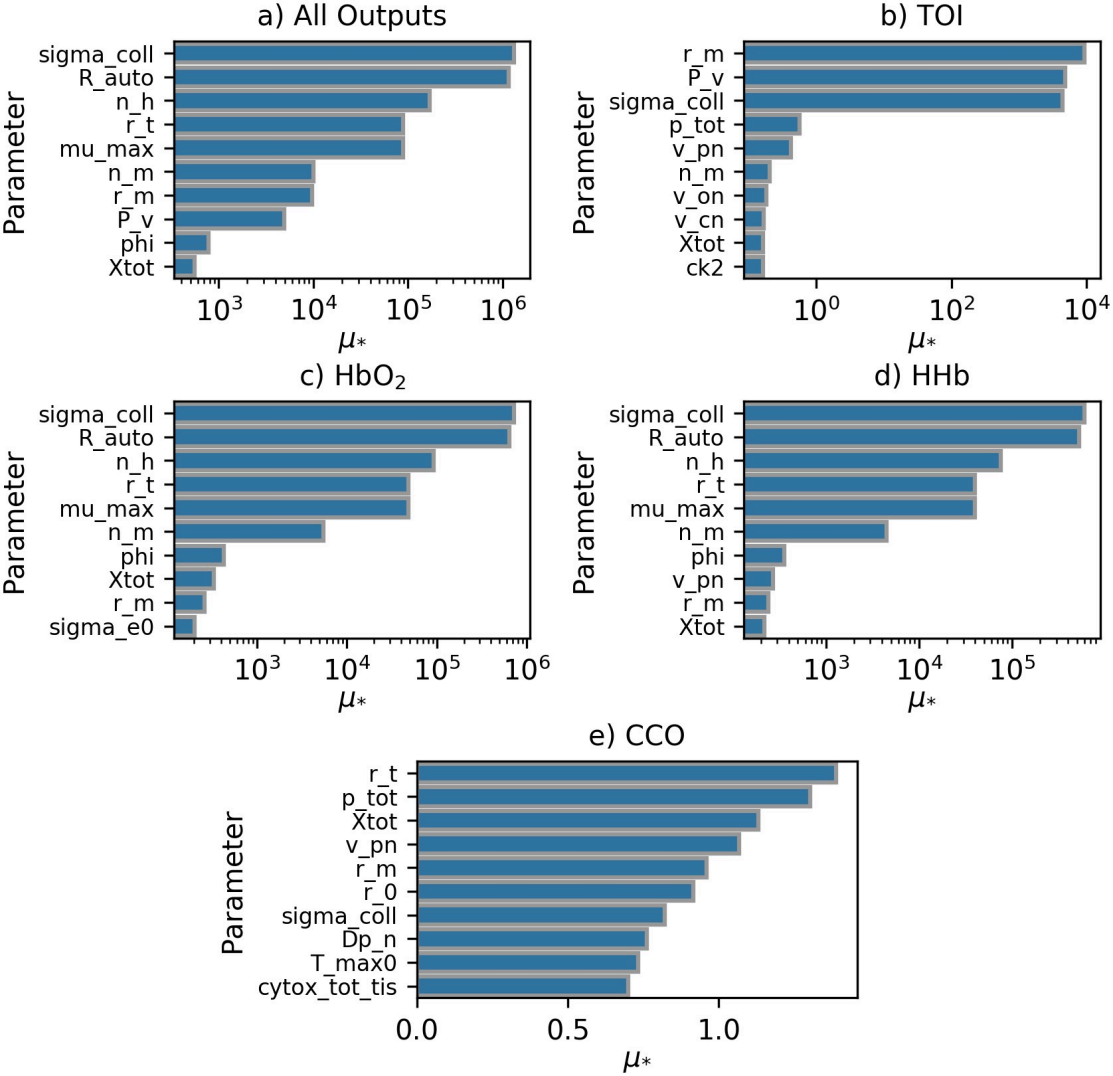
Sensitivity analysis across all outputs for simulated data set. Bar charts showing μ_*_ for the 10 most sensitive parameters across all model outputs, with values plotted on a log scale where appropriate. Distance used for calculation is the sum of *ε*_*SBTP*_ across all model outputs. All outputs except cytochrome-*c*-oxidase alone have μ_*_ values that vary on a logarithmic scale. Fig 7a shows results for all outputs combined, Fig 7b for TOI, Fig 7c for HbO_2_, Fig 7d for HHb and Fig 7e for CCO.

Sensitivity analysis was performed for the simulated healthy data set for the CCO, HbO_2_, HHb and TOI outputs. Fig 7 shows the sensitivity analysis results across all four outputs individually and for the outputs considered jointly. The results are plotted as bar charts, with sensitivity, as per the μ_*_ value, on the x-axis. The corresponding *σ* values for each parameter can be seen in S1 Fig.

Table 2 shows the selected parameters, their respective μ_*_ values and their definitions and default values. The total sensitivity analysis results, shown in Fig 7a, produced 10 parameters to be used in fitting the model. Sensitivity analysis based on individual outputs showed that different parameters were important for different outputs, with TOI, in Fig 7b being dominated by r_m, P_v and sigma_coll and oxyhaemoglobin, in Fig 7c, and deoxyhaemoglobin, in Fig 7d, dominated by sigma_coll and R_auto. Cytochrome-*c*-oxidase however showed levels of dependence that were similar across many parameters, as seen in Fig 7e, with μ_*_ values falling within a range of 0.7. For all individual outputs and the combined output, only Xtot, r_m and sigma_coll were within the 10 most sensitive in all cases.

**Table 2:** Sensitivity analysis results for simulated data, including each selected parameter’s definition and default value. * See [6] and [3] for a full explanation of this parameter and the stimulus *μ*.

Parameter	μ_*_	Definition	Default value
sigma_coll	1.32 × 10^6^	Pressure at which blood vessels collapse.	62.79 mmHg
R_auto	1.16 × 10^6^	Autoregulatory reactivity to oxygen.	1.5
n_h	1.68 × 10^5^	Hill coefficient for oxygen dissociation from haemoglobin.	2.5
r_t	8.82 × 10^4^	Radius in the muscular tension relationship.	0.018 cm
mu_max	8.82 × 10^4^	Upper bound for the transformed stimulus μ*.	1
n_m	9.97 × 10^3^	Exponent in the muscular tension relationship.	1.83
r_m	9.77 × 10^3^	Vessel radius at which muscular tension is maximal.	0.027 cm
P_v	4.88 × 10^3^	Venous blood pressure.	4 mmHg
phi	7.85 × 10^2^	Oxygen concentration at half-maximal saturation.	0.036 mM
Xtot	5.54 × 10^2^	Total concentration of haemoglobin O_2_ binding sites in blood.	9.1 mM

#### Experimental data

**Fig 8:**
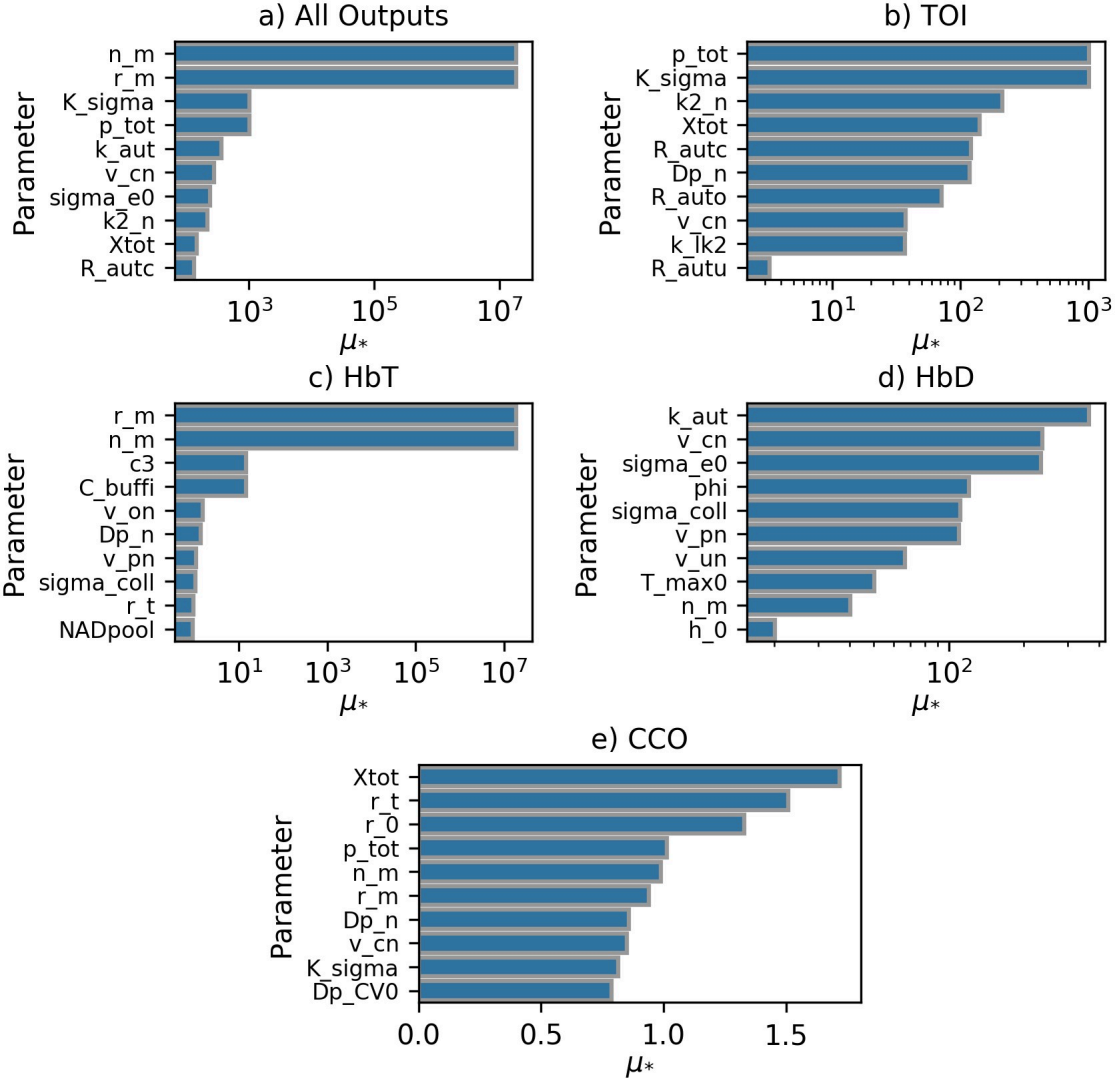
Sensitivity analysis across all outputs for experimental data set. Barplots showing μ_*_ values for the 10 most sensitive parameters across all model outputs, with the x-axis plotted using a log scale where appropriate. Distance used for calculation is the sum of *ε_SBTP_* across all model outputs. Fig 8a shows results for all outputs combined, Fig 8b for TOI, Fig 8c for HbT, Fig 8d for HbD and Fig 8e for CCO.

Sensitivity analysis was undertaken on the experimental dataset to determine the parameters to be fit. Table 3 shows the selected parameters, their respective μ_*_ values and their definitions and default values. Fig 8 shows the results across all outputs. The corresponding *σ* values for each parameter can be seen in S2 Fig. When considering all outputs jointly, the effect of n_m and r_m is significantly larger than all other parameters, but when looking at the individual outputs it’s clear that the other parameters are still important, but the magnitude of the impact n_m and r_m have on the overall variability is drastically larger.

**Table 3:** Sensitivity analysis results for experimental data, including each selected parameter’s definition and default value. *See [6] and [3] for a full explanation of this parameter and the stimulus *μ*. ^†^This is the arterial PaCO_2_ input put through a first order filter to simulate varying time response and is typically the same as arterial PaCO_2_. For more information see [3].

Parameter	μ_*_	Definition	Default Value
n_m	1.87 × 10^7^	Exponent in the muscular tension relationship.	1.83
r_m	1.87 × 10^7^	Vessel radius at which muscular tension is maximal.	0.027 cm
K_sigma	1.01 × 10^3^	Parameter controlling the sensitivity of *σ*_*e*_ to vessel radius *.	10
p_tot	6.29 × 10^3^	Total protons removed from the mitochondrial matrix by the three modelled electron transport reactions.	20
k_aut	3.65 × 10^2^	Parameter controlling overall functioning of autoregulatory response, with a value of 1 meaning intact autoregulation.	1
v_cn	2.76 × 10^2^	Normal filtered PaCO_2_^†^.	40 mmHg
sigma_e0	2.37 × 10^2^	Parameter in the elastic tension relationship.	0.1425 mmHg
k2_n	2.17 × 10^2^	Normal forward reaction rate for the reduction of a_3_.	3915.68 s^−1^
Xtot	1.48 × 10^2^	Total concentration of haemoglobin O_2_ binding sites in blood (4 times the haemoglobin concentration).	9.1 mM
R_autc	1.31 × 10^2^	Autoregulatory reactivity to carbon dioxide.	2.2

Unlike the simulated dataset, 9 of the top 10 most sensitive parameters have μ_*_ values between approximately 10 and 1000 which is significantly smaller than the range of the μ_*_ values for TOI in the simulated data.

Similarly, the most sensitive parameters for HbD fall within a very small range with no one parameter obviously determining the majority of the output’s behaviour. In contrast, the two most sensitive parameters for HbT, r_m and n_m, are approximately 10^6^ times larger than the third highest. As with the simulated data, μ_*_ values for CCO have much smaller values than all other outputs and fall within a range of 1.0. Unlike the simulated data, no parameters were sensitive across all individual and joint outputs.

#### Parameters

Whilst a full exploration of the parameters within the BrainSignals model is outside the scope of this paper, we advise the reader to look at the original publications [3, 6], and provide a brief overview of some of those identified as important here.

A number of the parameters identified as being important for the above datasets, such as R_auto and mu_max, are dimensionless parameters. They are often model specific parameters that cannot be directly measured and instead need to be considered in the context of their meaning within the model. For example, an increase in R_auto would mean that the autoregulatory response would become more sensitive to changes in oxygen concentration. In contrast, other parameters such as Xtot, which is four times the concentration of haemoglobin, are more easily measured in an experimental or clinical setting.

Some of the parameters identified as important are linked closely to the shape of the autoregulatory response of the model and its sensitivity to changes in model inputs. These are R_auto and mu_max in the simulated dataset, and v_on, v_un, R_autc and v_cn in the experimental dataset. As we are driving the model with a changing input, the identification of these parameters as important seems physiologically sensible. It should also be noted that, despite other parameters not directly controlling the autoregulation response, the interconnected and complex nature of the BrainSignals model means that other parameters may still have an impact on it indirectly, for example the parameter r_t controls the stifness of blood vessel walls, which is important in controlling blood flow during autoregulation.

More detailed information on the exact nature of these parameters and how they function within the BrainSignals model can be found in [6].

### Bayesian analysis

#### Simulated data

**Fig 9:**
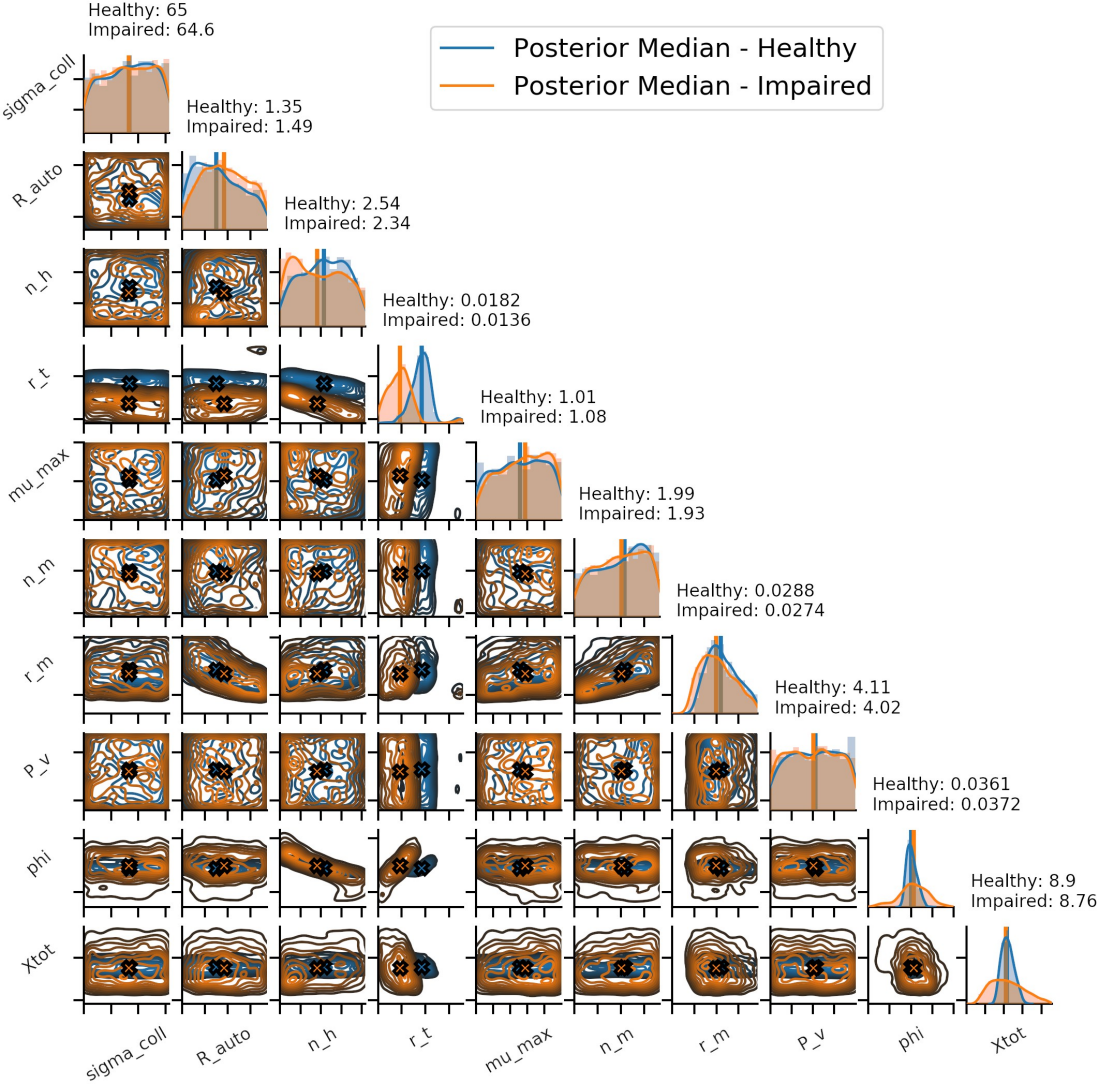
Comparison of posterior distributions for healthy and impaired simulated data. Fig 9 shows the posteriors for healthy and impaired data based on an acceptance rate of 0.01%. Posterior are shown over the full prior range as defined in S1 and S2 Tables.

**Fig 10:**
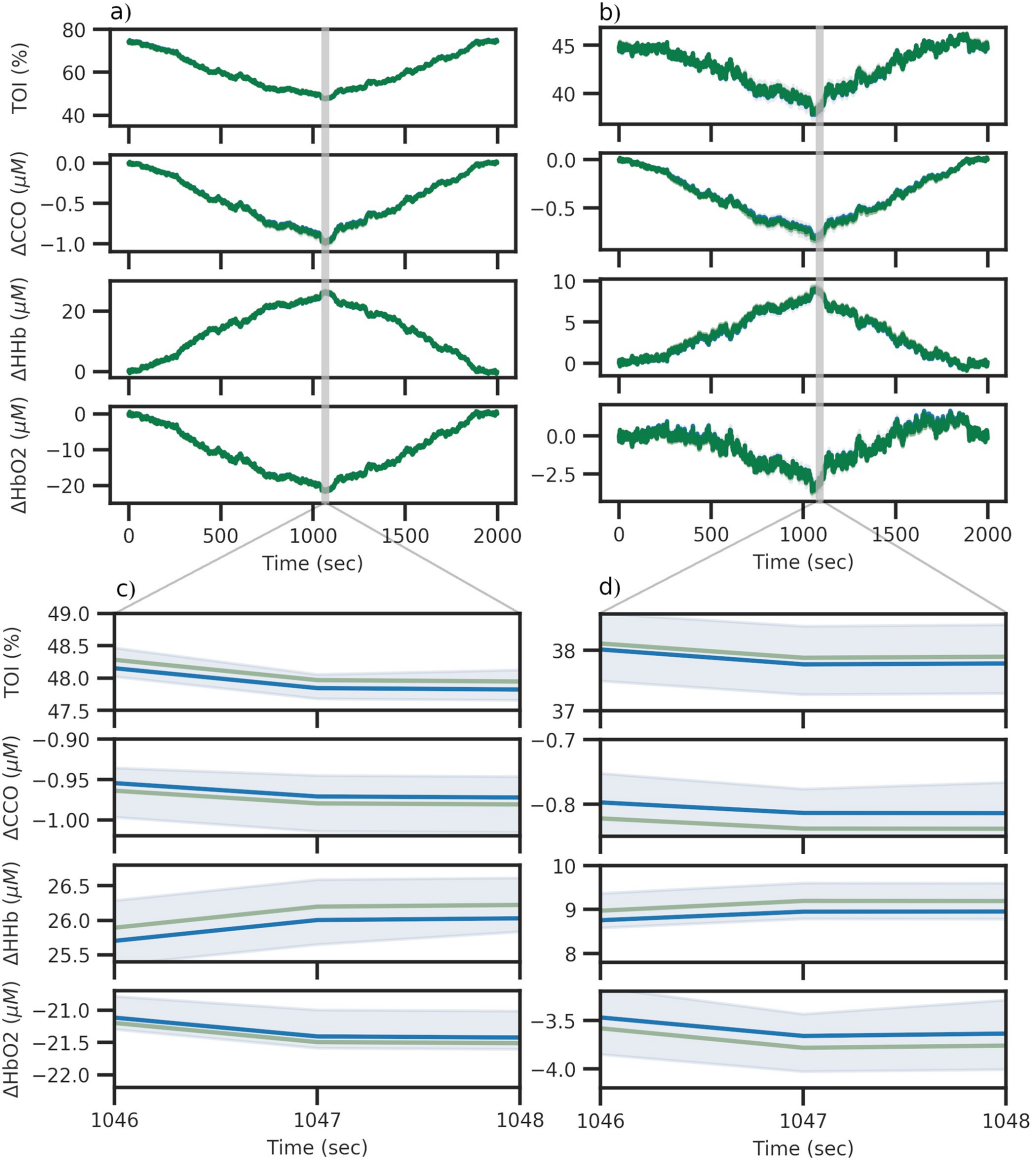
Comparison of predictions for healthy and impaired simulated data. Figures 10a and 10b show the predicted time series data from the healthy and impaired posteriors respectively. Each posterior was sampled 25 times and the resulting runs aggregated, with the median and 95% credible intervals plotted in dark blue and light blue respectively. Figures 10c and 10d show a zoomed in view of each output in order to show the credible interval of the posterior predictive distribution.

The BrainSignals model was fit to the simulated “healthy” dataset initially. The model was run for 10,000,000 different parameter combinations before determining that the posterior had been estimated sufficiently well, based on the quality of the posterior predictive distribution. The samples in the posterior were found to have 0.019170 ≤ *ε*_NRMSE_ ≤ 0.098098. Fig 9 shows this posterior distribution in blue. Xtot, phi and r_t show narrow marginal distributions whilst the others are much wider. Median values for all parameters are close to the model value, with R_auto showing a skew towards lower values in its marginal distribution that also leads to a median slightly lower than the model value. Fig 10a shows the posterior predictive distribution produced by sampling 25 times directly from the posterior, and shows a very good fit.

This healthy posterior was then used to define an impaired brain, as mentioned above. r_t was set to 0.013 mmHg and the model driven with the same inputs as the healthy simulation. This “impaired” dataset was then fit using the same approach as above, using the sensitivity analysis results. The model was run 30,000,000 different parameter combinations, with the increased run number required in order to sufficiently estimate the posterior. With an acceptance rate of 0.01%, a posterior was produced based on 3000 samples having 0.019170 ≤ *ε*_NRMSE_ ≤ 0.267152. Despite the higher error values as compared to the healthy data, the resulting fit was still deemed very good. Fig 9 shows this posterior in orange and Fig 10b shows the time series generated by sampling 25 times directly from this posterior. Xtot, phi and r_t show marginal distributions that are narrower than the others, but wider than those seen in the healthy posterior. All parameters have median values close to the value set in the model. A separation between the healthy r_t and impaired r_t marginal distributions is clearly visible.

Fig 10c and 10d show a zoomed in view of each output in order to show the 95% credible interval of the posterior predictive distribution. This is not clearly visible on the full trace as it is reasonably small.

Sections S3 to S10 Figs of the supplementary material show a number of different statistical analyses of the results for the healthy and impaired datasets respectively. These are all posterior predictive checks, where the posterior predictive distribution is used to produce a number of statistical results that can be used to assess the quality of the model fit. S3 Fig shows the autocorrelation of both the posterior predictive and observed data as a function of lag for each signal in the healthy data. S4 Fig shows the distribution of the residuals for each signal in the healthy dataset, with the mean and standard deviation drawn on. Q-Q plots for these distributions are shown in S5 Fig and are used to assess the normality of the residuals. S6 Fig shows the prior and posterior distributions for each parameter along with the Kullback-Leibler Divergence for each of these, giving us a sense of how much information was gained when moving from prior to posterior distribution. S7, S8, S9 and S10 Figs show these same posterior predictive checks but for the impaired distribution.

The autocorrelation plots in S3 and S7 Figs show that the autocorrelation of the posterior predictive distribution and the observed data match closely across all lag values for both healthy and impaired datasets. Looking at the distributions of the residuals for each signal in S4 and S8 Figs we can see that the residuals for TOI appear to be normally distributed, whilst the other three signals all show generally symmetric but leptokurtic distributions. The Q-Q plots in S5 and S9 Figs confirm this, suggesting that the residuals across all signals are generally normally distributed, with some slight differences at the highest and lowest quantiles. Residuals for HHb and CCO both appear to be generally more leptokurtic than both TOI and HbO_2_.

#### Experimental data

When approaching the experimental data, the criteria for a good fit were different to those in the simulated dataset. With the simulated dataset, any parameters not chosen for fitting would have the same value during the fitting process as during the generation of the simulated dataset. In the experimental data however, it is almost certain that the default values of any parameters not chosen for fitting would not have the exact same value as their biological, real-world analogue. As a result, instead of looking for a perfect fit, we instead look for qualitative behaviours to be reproduced, such as the periodic increase and decrease in output values due to the repeated hypoxia challenges.

**Fig 11:**
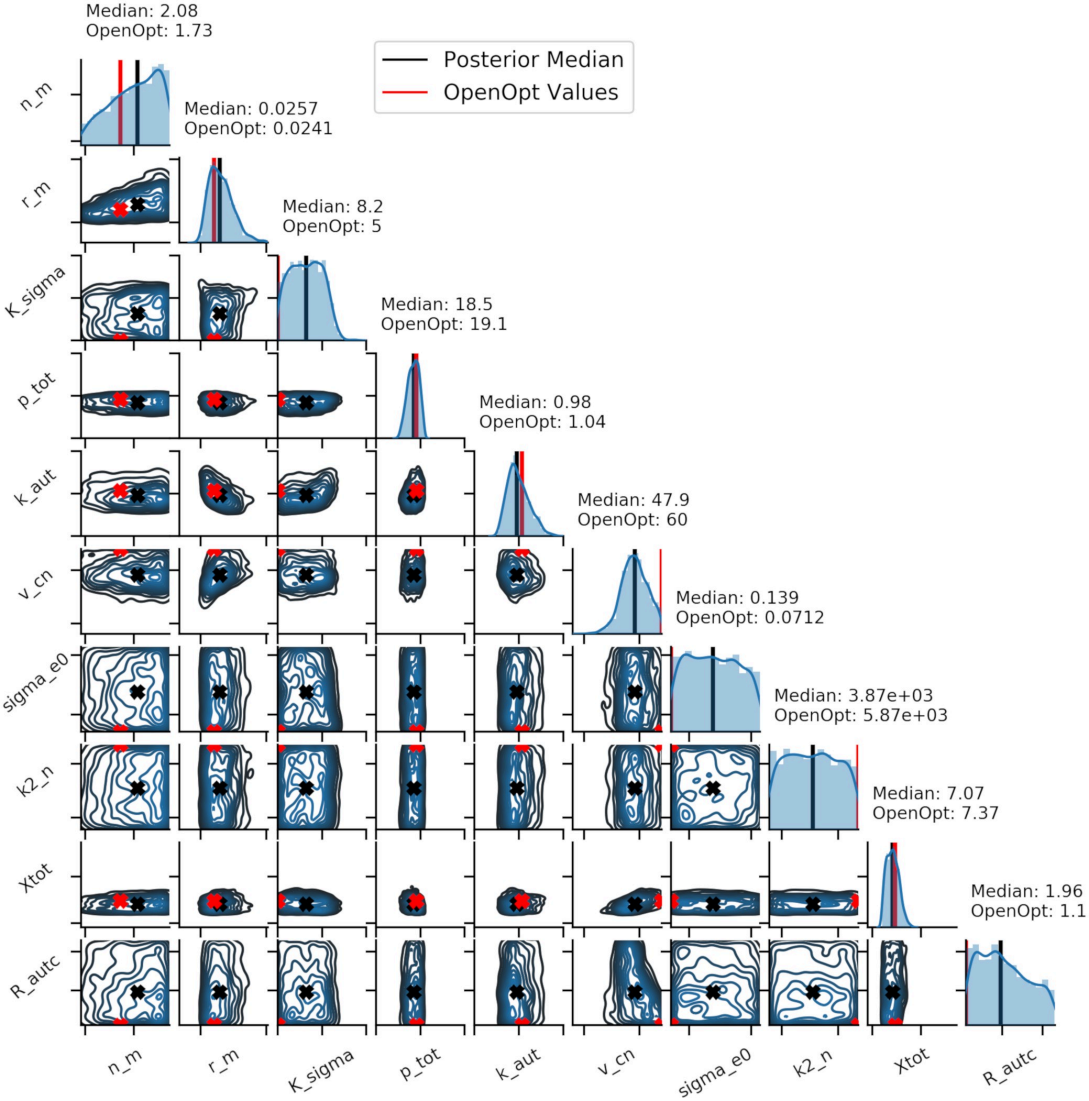
Posterior distributions for the experimental data set. Fig 11 shows the posterior distribution for the experimental data set, based on an acceptance rate of 0.01%. The posterior median is shown in black and the OpenOpt predicted value is shown in red. Posterior are shown over the full prior range as defined in S3 Table.

**Fig 12:**
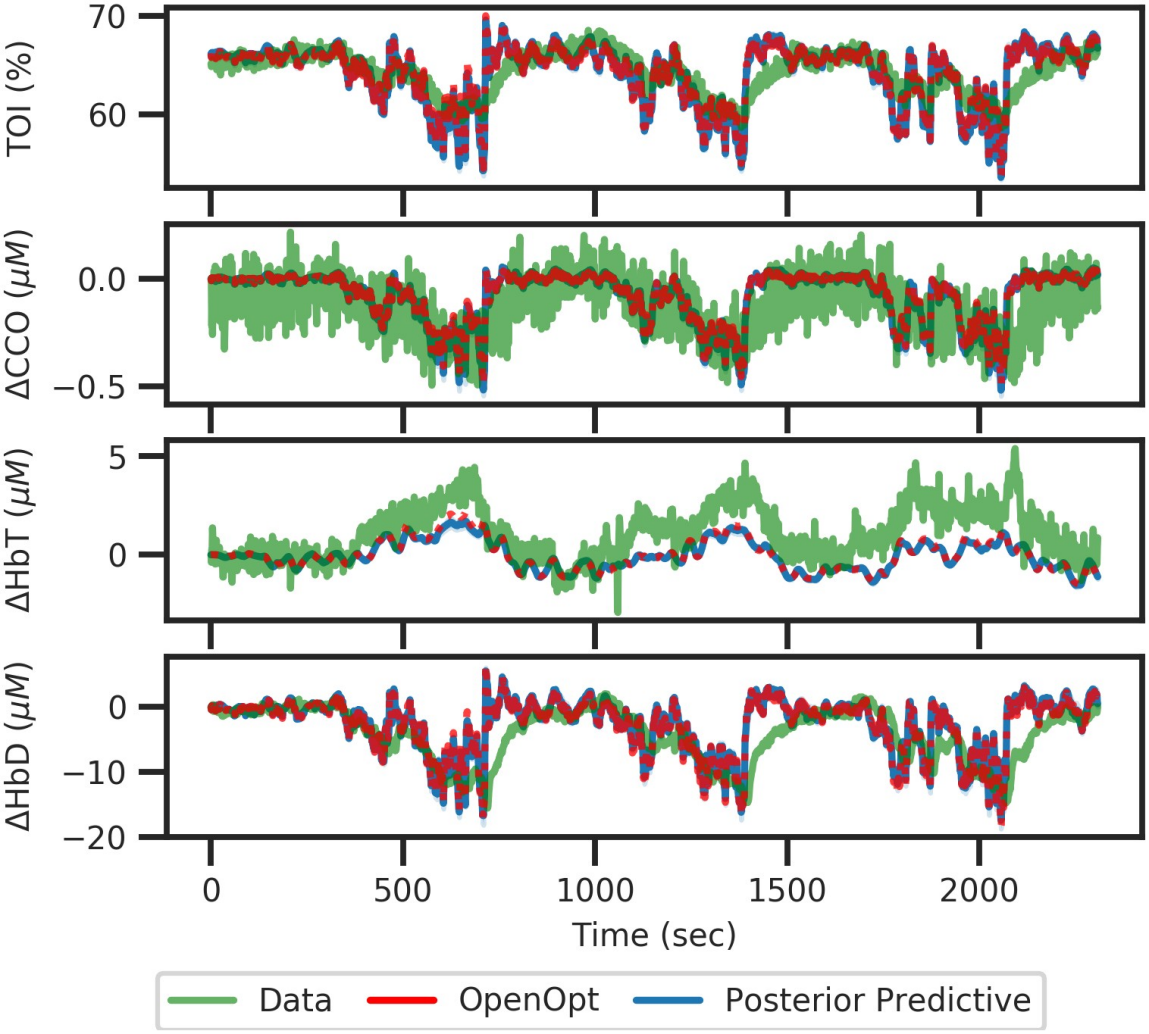
Predicted fits for the experimental data set. Fig 12 shows the predicted time series for all output based on the posterior shown in Fig 11. The posterior was sampled 25 times with the resulting time series aggregated, with the median and 95% credible intervals plotted in dark and light blue respectively. Overall behaviour is reflected in the predicted trace, with 3 distinct periods of hypoxia visible as periodic behaviour within all signals. The fit obtained using OpenOpt is shown in red.

The fitting process required 20,000,000 parameter combinations before a satisfactory fit was obtained, and with an acceptance rate of 0.01% the posterior in Fig 11 consisted of 2000 samples with 0.778492 ≤ *ε*_NRMSE_ ≤ 0.802900. The model was also fit using the previous OpenOpt method, and the values obtained from that are also shown for comparison. We can see that for parameters with reasonably well defined posterior, the OpenOpt values and the posterior median are reasonably close, but for those showing a wider distribution, the OpenOpt value can vary massively from the posterior median. For sigma_e0 and k2_n the OpenOpt value is at one extreme end of the prior range, whilst the median remains central due to the distribution being uniform. Fig 12 shows the predicted time series for all outputs based on the posterior shown in Fig 11. The posterior was sampled 25 times with the resulting time series aggregated, with the median and 95% credible intervals plotted. Overall behaviour is reflected in the predicted trace, with 3 distinct periods of hypoxia visible as periodic behaviour within all signals. Shown in green is the fit obtained using the OpenOpt method, which has an error *ε*_NRMSE_ = 0.77518. It is clear that both methods are able to achieve similar fits, but the Bayesian method provides more information about the space of possible parameter combinations and the resultant uncertainty in fitted model output.

As with the simulated datasets, we have produced posterior predictive checks to assess the fit of the model to the data. These are shown in Sections S11 to S14 Figs of the supplementary material. S11, S12, S13 and S14 Figs show the autocorrelation comparisons, distribution of residuals, Q-Q plots and prior-posterior comparison plots respectively.

The autocorrelation plot in S11 Fig show that the autocorrelation of the posterior predictive distribution and the observed data generally match across all lag values, with the same shape seen in both plots, but with a slight difference in the magnitude of the autocorrelation. The distributions of residuals in S12 Fig all appear to be relatively normally distributed, with TOI, CCO and HbD showing a mean close to zero. HbT however has a mean noticeably less than zero, which is due to the simulation predicting generally lower values than in the observed data, as seen in Fig 12. These results are more clearly seen in S13 Fig, which suggests that the residuals for all signals are generally normal, with some bimodality in the HbT distribution and a significant amount of positive skew in the HbD distribution.

## Discussion

In this work we have introduced a new Bayesian analysis for interpretation of the BrainSignals models. The process was tested and used to analyse two simulated datasets and one experimental data set. The Bayesian approach provides us with complete information about the parameter space and takes into account the prior information we have about physiological parameters via the proposal distribution, *p*(*θ*), which allows us to simulate distributions of input parameters. Both of these factors are extremely important when drawing physiological conclusions from any parameter estimates.

Using the posterior predictive checks in sections S3 to S14 Figs we have shown that the Bayesian method is able to produce good fits for a range of different datasets, including both overly simplistic test cases, with just a single parameter change, and real world measured data which contains inherently more complexity than simulated data. It can also be seen from direct observation of the posterior predictive distribution in Fig 12 that the Bayesian method is able to provide a fit equally as good as the previous OpenOpt method.

We have shown how the method can be used to define healthy and impaired parameter spaces, as shown with the simulated datasets, and how for some parameters these spaces may overlap. We have also shown how the new Bayesian approach provides more information about the parameter space than the previous OpenOpt maximum likelihood method. Looking at only the healthy data set, the parameters sigma_coll, P_v and mu_max all have marginal posteriors with a median at the default value set in the model, but with distributions that cover the entirety of the prior distribution initially set. Determining that a parameter’s posterior distribution is not tightly constrained is important when drawing physiological conclusions from the model fitting process. This is seen even more clearly in the direct comparison between prior and posterior distributions for these parameters in S6 Fig and in their K-L divergence values of 0.00971 nats, 0.0109 nats and 0.014 nats respectively. If we compare these to the plots and K-L divergence values for phi and Xtot, which have values of 1.38 nats and 1.04 nats respectively, it is clear that the Bayesian process provides significantly more information than the previous OpenOpt method, both in terms of producing posteriors that have significant information gains compared to prior distributions, and in identifying parameters where a good fit is produced despite minimal information gain. Using only sensitivity analysis and OpenOpt would not provide this extra insight.

This is seen more clearly when looking at the experimental data. Many of the parameters show relatively narrow marginal posteriors, but sigma_e0 and k2_n, which were both identified as important by the original sensitivity analysis, are both shown to have wide distributions, suggesting insensitivity within the prior range. The previous OpenOpt method produces an almost identical fit as the Bayesian approach but provides significantly less information about the parameter space. For sigma_e0, k2_n, v_cn, R_autc and k_sigma the OpenOpt values fall outside of the interquartile range of the posterior distribution, yet produce equivalent model simulations. If considering the OpenOpt estimate alone, it would be simple to draw the conclusion that these parameters have shifted away from the default ‘healthy’ value, showing some sort of physiological change during the hypoxia challenge. However, when we look at the posterior obtained through the Bayesian method, the median value is close to the default value and in fact parameter values across the entire prior range produce similar results. As a result we can instead say that for this data the model is insensitive to these parameters, with a median value that would be considered ‘healthy’. Again, if we look at the comparison between prior and posterior distributions and consider the K-L divergence for each parameter in S14 Fig, it is clear to see where we have and have not gained information through the use of the Bayesian process.

There are a number of other methods that can also be used to explore and define the parameter space. The previously used maximum likelihood based method, for example, can provide estimates and confidence limits of parameter values, but under the assumption that the maximum likelihood estimator is normally distributed around the maximum. It may also be possible to use a profile likelihood [40], but whilst this will provide information about the distribution of the parameter space without assuming normality, it is computationally expensive and does not take into account prior information about the parameters.

It is acknowledged that the Bayesian approach is not without its own limitations. Historically, non-trivial problems were not solvable analytically due to the high dimensional integrals required. However, with the relatively recent availability of more computational power, a number of algorithms and approaches are now available that allow these problems to be approximated. This has seen increased uptake of Bayesian approaches within the fields of systems biology and genetics, where the inherently complex models and noisy data that these fields involve are particularly well suited to being analysed through the Bayesian approach. As long as a statistical model can be used to relate the relevant quantities, Bayesian inference can be used to give full probabilistic information on all unobserved model variables.

One of the main drawbacks to this method is that the number of model runs required to have sufficient samples in the posterior may be prohibitively high, especially where the tolerance is low or the prior distribution is very different from the posterior distribution.

**Fig 13:**
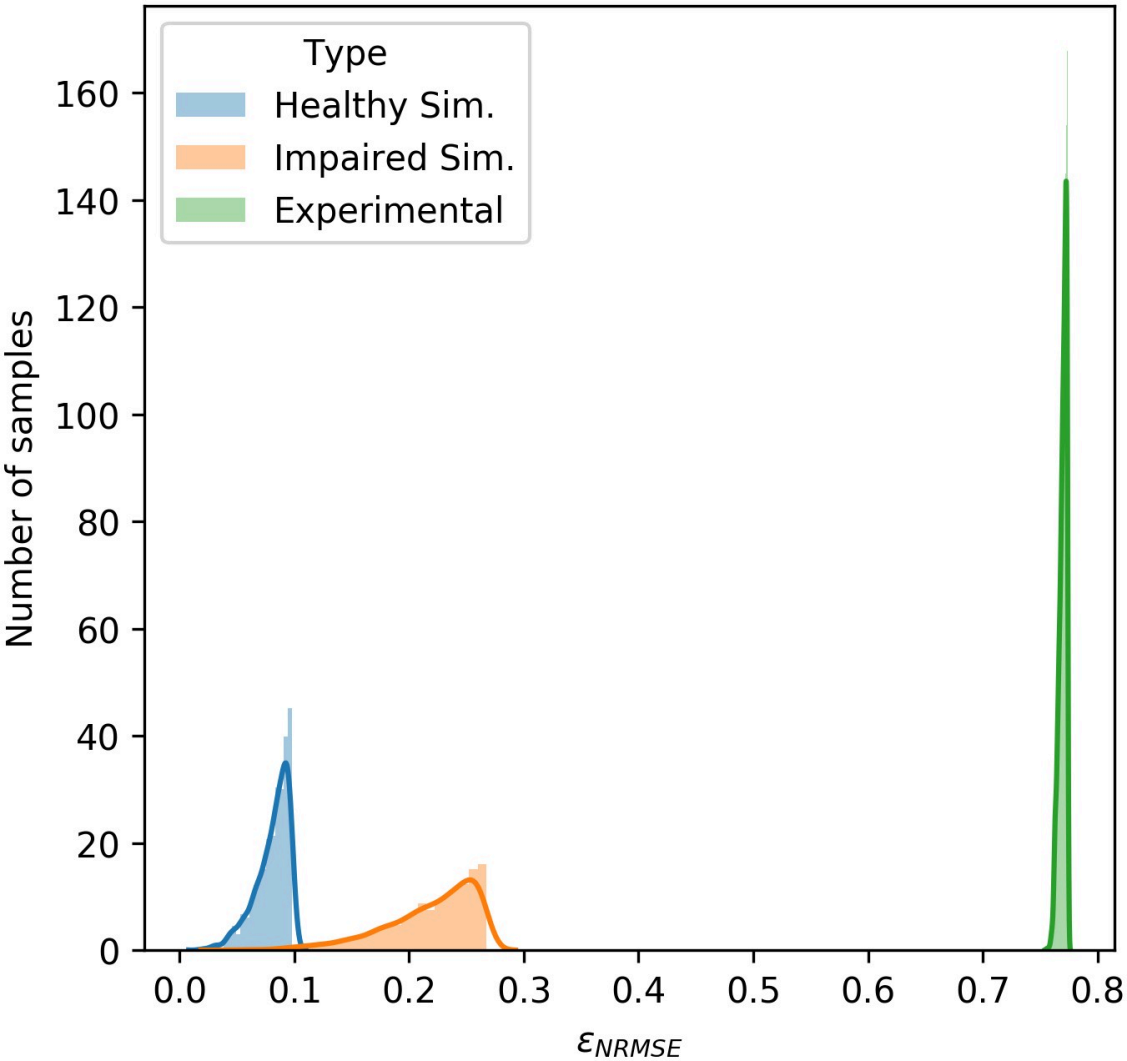
Distribution of *ε*_NRMSE_ values for the posteriors of each dataset. It can be seen here that the three datasets had very different distributions *ε*_NRMSE_ values for the samples that made up their respective posteriors. Despite this, the posterior predictive distributions for all datasets were good fits.

This requirement for a large number of simulations for a reliable posterior is seen in all of the datasets used here. For the simulated ‘healthy’ data, the model sampled 10,000,000 parameter combinations in order to achieve the obtained fit. In contrast, to fit the ‘impaired’ simulated data the model required 30,000,000 parameter combinations to be sampled and for the same acceptance rate the accepted samples had generally higher *ε*_NRMSE_ values. Finally, the experimental data was only able to obtain a good posterior after being sampling 20,000,000 parameter combinations and all *ε*_NRMSE_ values were significantly above those seen in the simulated datasets. This is clearly visible in Fig 13, where the distribution of *ε*_NRMSE_ values for each posterior are clearly very different. This figure clearly highlights the variance in both the error values that define a ‘good’ fit and the number of samples required for a reliable posterior distribution for different datasets.

It should be noted that all of the obtained posterior distributions produce what are considered good fits, with those obtained for the simulated datasets far more accurate than we would ever expect to achieve when fitting experimental data. When looking at the experimental data in particular, despite the *ε*_NRMSE_ values being much higher than in the simulated data, the obtained fit captures all important behaviour and phenomena, with three clear hypoxia events visible in the inferred data trace.

More efficient methods of ABC, may alleviate the problem of requiring so many model runs to obtain good posteriors. An approach based on MCMC is more efficient than ABC REJ but the chain may become stuck in regions of low probability for long periods of time [41]. In order to deal with this problem and also the disadvantages of the rejection algorithm, an approach based on sequential Monte Carlo (ABC SMC) [38] was first proposed by Sisson et al. [42], as well as Beaumont et al. [43] and Cappé et al. [44]. In this approach, a number of sampled parameter values, known as *particles*, are sampled from the prior distribution and then propagated through a number of intermediate distributions before reaching a final target distribution. The tolerance for each successive distribution is smaller than the previous, allowing them to evolve towards the target posterior. Additionally, for a sufficiently large number of particles, the problem in MCMC of getting stuck in areas of low probability can be avoided. Developing the BayesCMD framework to use an ABC SMC approach is a key focus for future work.

### Conclusion

We have outlined how this new Bayesian framework for model analysis can be used with models of brain haemodynamics to extract information from physiological data. A more comprehensive picture of the parameter space is obtained, allowing physiological conclusions to be based on a broader picture. This is most clearly seen in the experimental data, where point estimates suggested that the values for a number of parameters had changed significantly during fitting, whilst the Bayesian method showed that the parameters were defined by a broad, roughly uniform distribution. We have also shown, through the use of data simulated from the BrainSignals model in healthy and impaired states, how the Bayesian approach allows us to better distinguish different parameter spaces. Finally, whilst we have focussed on using the BrainSignals model here, any model that can be written in a format compatible with BCMD can use this method to estimate model parameters.

A major interest within our research group is to use these models and approaches to understand and investigate further our novel measures of brain tissue physiology and metabolism and how they are linked to brain injury [45, 46]. In particular, we are interested in neonatal hypoxic ischaemic injury. The Bayesian approach provides a better representation of the parameter space and can inform a better distinction between different brain states, such as between a mild and severe injury. The method will also be adapted to use more efficient methods of parameter estimation, such as ABC SMC, reducing the number of model runs required to obtain a given tolerance.

## Supporting information

S1 Table Table of posterior and prior distribution information for healthy simulated data. (PDF)

S2 Table Table of posterior and prior distribution information for impaired simulated data. (PDF)

S3 Table Table of posterior and prior distribution information for experimental data. (PDF)

S1 Fig Bar charts of the *σ* values from the sensitivity analysis of the simulated data. (PDF)

S2 Fig Bar charts of the *σ* values from the sensitivity analysis of the experimental data. (PDF)

S3 Fig Autocorrelation of posterior predictive and the observed data for the simulated healthy data. (PDF)

S4 Fig Distributions of residuals for the simulated healthy data. (PDF)

S5 Fig Q-Q plots of residuals for the simulated healthy data. (PDF)

S6 Fig Comparison of marginal posterior and prior distributions for the simulated healthy data. (PDF)

S7 Fig Autocorrelation of posterior predictive and the observed data for the simulated impaired data. (PDF)

S8 Fig Distributions of residuals for the simulated impaired data. (PDF)

S9 Fig Q-Q plots of residuals for the simulated impaired data. (PDF)

S10 Fig Comparison of marginal posterior and prior distributions for the simulated impaired data. (PDF)

S11 Fig Autocorrelation of posterior predictive and the observed data for the experimental data. (PDF)

S12 Fig Distributions of residuals for the experimental data. (PDF)

S13 Fig Q-Q plots of residuals for the experimental data. (PDF)

S14 Fig Comparison of marginal posterior and prior distributions for the experimental data. (PDF)
