# Supplementary material for: A Bayesian framework for the analysis of systems biology models of the brain
Source: PLoS Comput Biol. 2019 Apr 26;15(4):e1006631. doi: 10.1371/journal.pcbi.1006631 (PMC6505968; doi:10.1371/journal.pcbi.1006631)
Supplement: S1 Table — (PDF) [file pcbi.1006631.s001.pdf]

S1 Table. Table of posterior and prior distribution information for healthy simulated data.

	Posterior				Prior	
Parameter	Median	Lower Quartile	Upper Quartile	IQR	Prior Min.	Prior Max.
sigma_coll	65.04	49.4	79.87	30.47	31.395	94.185
R_auto	1.353	1.053	1.721	0.6679	0.75	2.25
n_h	2.541	1.988	3.079	1.09	1.25	3.75
r_t	0.01822	0.01692	0.01938	0.002461	0.009	0.027
mu_max	1.014	0.7679	1.26	0.4922	0.5	1.5
n_m	1.992	1.493	2.369	0.876	0.915	2.745
r_m	0.02879	0.02568	0.03273	0.007059	0.0135	0.0405
P_v	4.112	3.095	5.067	1.972	2	6
phi	0.03609	0.03474	0.03775	0.003013	0.018	0.054
Xtot	8.898	8.463	9.406	0.943	4.55	13.65

Posterior and prior distribution information for healthy simulated data. Posterior distribution values are given to 4 significant figures. Prior range values are given as their exact values.
