# Supplementary material for: A Bayesian framework for the analysis of systems biology models of the brain
Source: PLoS Comput Biol. 2019 Apr 26;15(4):e1006631. doi: 10.1371/journal.pcbi.1006631 (PMC6505968; doi:10.1371/journal.pcbi.1006631)
Supplement: S2 Table — (PDF) [file pcbi.1006631.s002.pdf]

S2 Table. Table of posterior and prior distribution information for impaired simulated data.

	Posterior				Prior	
Parameter	Median	Lower Quartile	Upper Quartile	IQR	Prior Min.	Prior Max.
sigma_coll	64.58	48.86	79.79	30.93	31.395	94.185
R_auto	1.488	1.183	1.831	0.6479	0.75	2.25
n_h	2.337	1.742	3.014	1.272	1.25	3.75
r_t	0.0136	0.01183	0.01508	0.00325	0.009	0.027
mu_max	1.076	0.8283	1.289	0.4609	0.5	1.5
n_m	1.928	1.471	2.344	0.873	0.915	2.745
r_m	0.02738	0.02372	0.03174	0.00802	0.0135	0.0405
P_v	4.022	2.966	4.964	1.998	2	6
phi	0.03722	0.03349	0.04122	0.007732	0.018	0.054
Xtot	8.764	7.534	10.18	2.646	4.55	13.65

Posterior and prior distribution information for impaired simulated data. Posterior distribution values are given to 4 significant figures. Prior range values are given as their exact values.
