# Supplementary material for: A Bayesian framework for the analysis of systems biology models of the brain
Source: PLoS Comput Biol. 2019 Apr 26;15(4):e1006631. doi: 10.1371/journal.pcbi.1006631 (PMC6505968; doi:10.1371/journal.pcbi.1006631)
Supplement: S3 Table — (PDF) [file pcbi.1006631.s003.pdf]

S3 Table. Table of posterior and prior distribution information for experimental data.

	Posterior				Prior	
Parameter	Median	Lower Quartile	Upper Quartile	IQR	Prior Min.	Prior Max.
n_m	2.078	1.645	2.446	0.8011	0.915	2.745
r_m	0.02574	0.02342	0.02841	0.004988	0.0135	0.0405
K_sigma	8.195	6.701	9.663	2.961	5	15
p_tot	18.48	17.45	19.4	1.943	10	30
k_aut	0.9799	0.9073	1.074	0.167	0.5	1.5
v_cn	47.88	43.93	52.13	8.199	20	60
sigma_e0	0.1386	0.1043	0.1739	0.06966	0.07125	0.2137
k2_n	3870	2903	4822	1919	1957.83	5873.52
Xtot	7.067	6.589	7.555	0.9663	4.55	13.65
R_autc	1.955	1.516	2.517	1.001	1.1	3.3

Posterior and prior distribution information for experimental data. Posterior distribution values are given to 4 significant figures. Prior range values are given as their exact values.
