# Supplementary material for: A Bayesian framework for the analysis of systems biology models of the brain
Source: PLoS Comput Biol. 2019 Apr 26;15(4):e1006631. doi: 10.1371/journal.pcbi.1006631 (PMC6505968; doi:10.1371/journal.pcbi.1006631)
Supplement: S2 Fig — (PDF) [file pcbi.1006631.s005.pdf]

S2 Fig. Bar charts of the σ values from the sensitivity analysis of the experimental data.

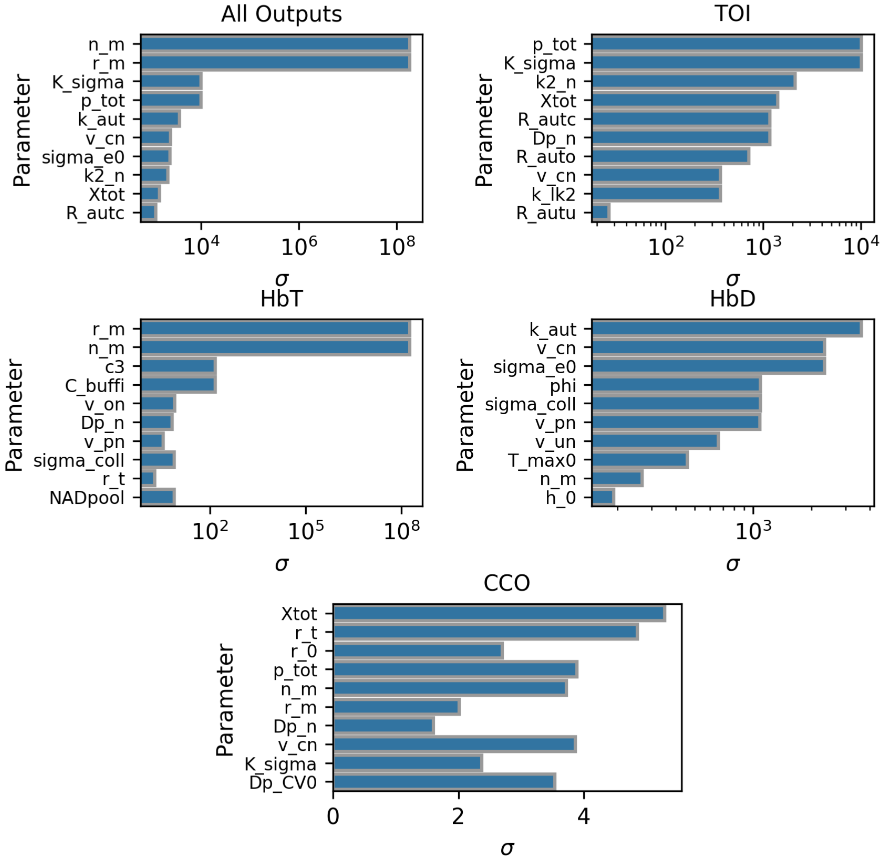

σ values for each signal as per the sensitivity analysis. Shown are the σ values for each of the 10 most sensitive parameters in each signal, as per μ_*, for the experimental dataset.
