# Supplementary material for: A Bayesian framework for the analysis of systems biology models of the brain
Source: PLoS Comput Biol. 2019 Apr 26;15(4):e1006631. doi: 10.1371/journal.pcbi.1006631 (PMC6505968; doi:10.1371/journal.pcbi.1006631)
Supplement: S4 Fig — (PDF) [file pcbi.1006631.s007.pdf]

S4 Fig Distributions of residuals for the simulated healthy data.

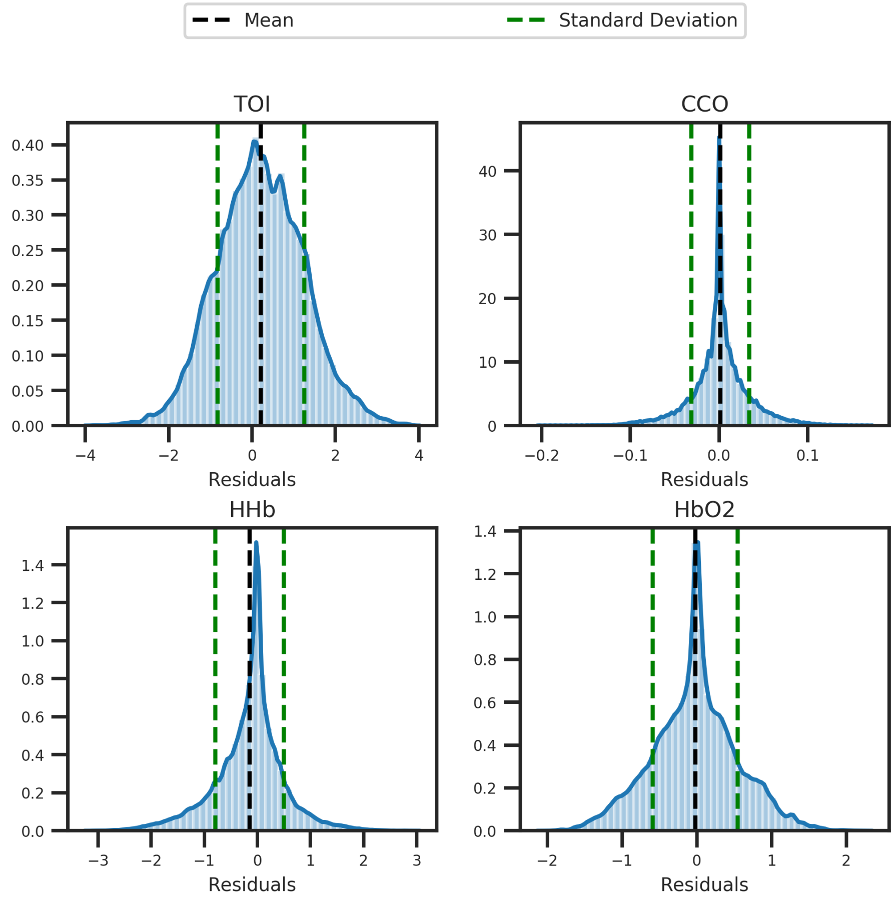

Distributions of residuals. Distributions of the residuals for each signal. Mean and standard deviation of each plot are indicated by black and green lines respectively.
