# Supplementary material for: A Bayesian framework for the analysis of systems biology models of the brain
Source: PLoS Comput Biol. 2019 Apr 26;15(4):e1006631. doi: 10.1371/journal.pcbi.1006631 (PMC6505968; doi:10.1371/journal.pcbi.1006631)
Supplement: S9 Fig — (PDF) [file pcbi.1006631.s012.pdf]

**S9 Fig Q-Q plots of residuals for the simulated impaired data.**

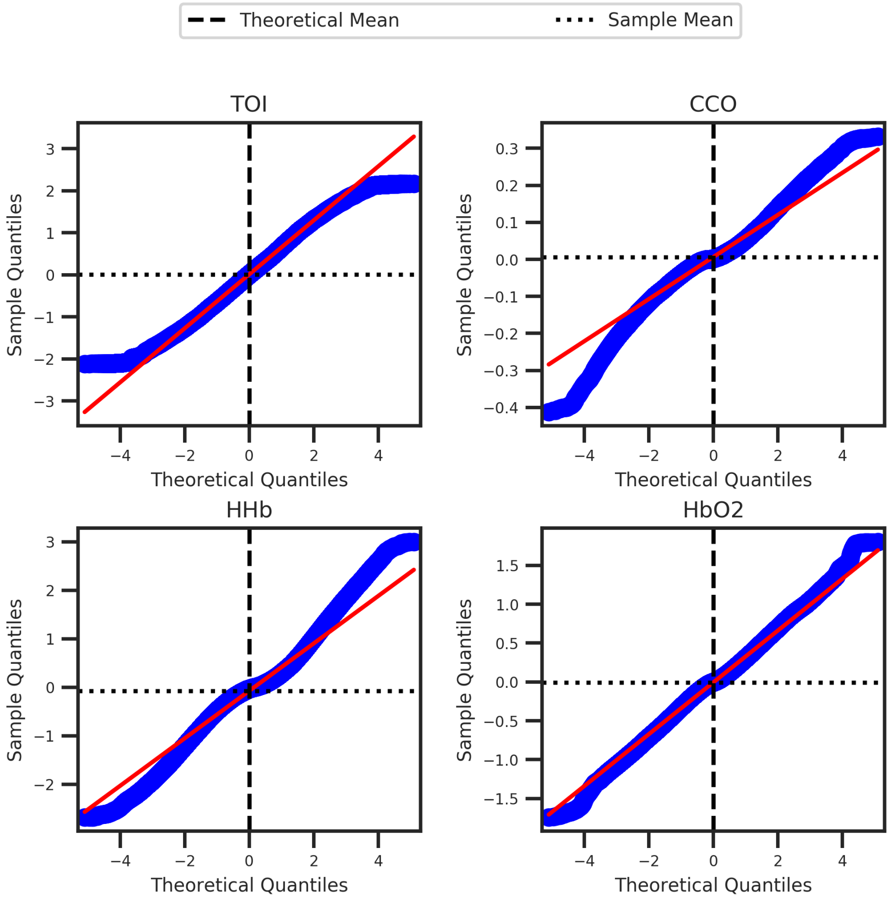


**Q-Q plot of residuals.** Q-Q plots looking at the residuals for each signal. The plots use a standardised diagonal line, where the expected order statistics are scaled by the standard deviation of the sample residuals and have the mean added to them.
