# Supplementary material for: A Bayesian framework for the analysis of systems biology models of the brain
Source: PLoS Comput Biol. 2019 Apr 26;15(4):e1006631. doi: 10.1371/journal.pcbi.1006631 (PMC6505968; doi:10.1371/journal.pcbi.1006631)
Supplement: S11 Fig — (PDF) [file pcbi.1006631.s014.pdf]

S11 Fig Autocorrelation of posterior predictive and the observed data for the experimental data.

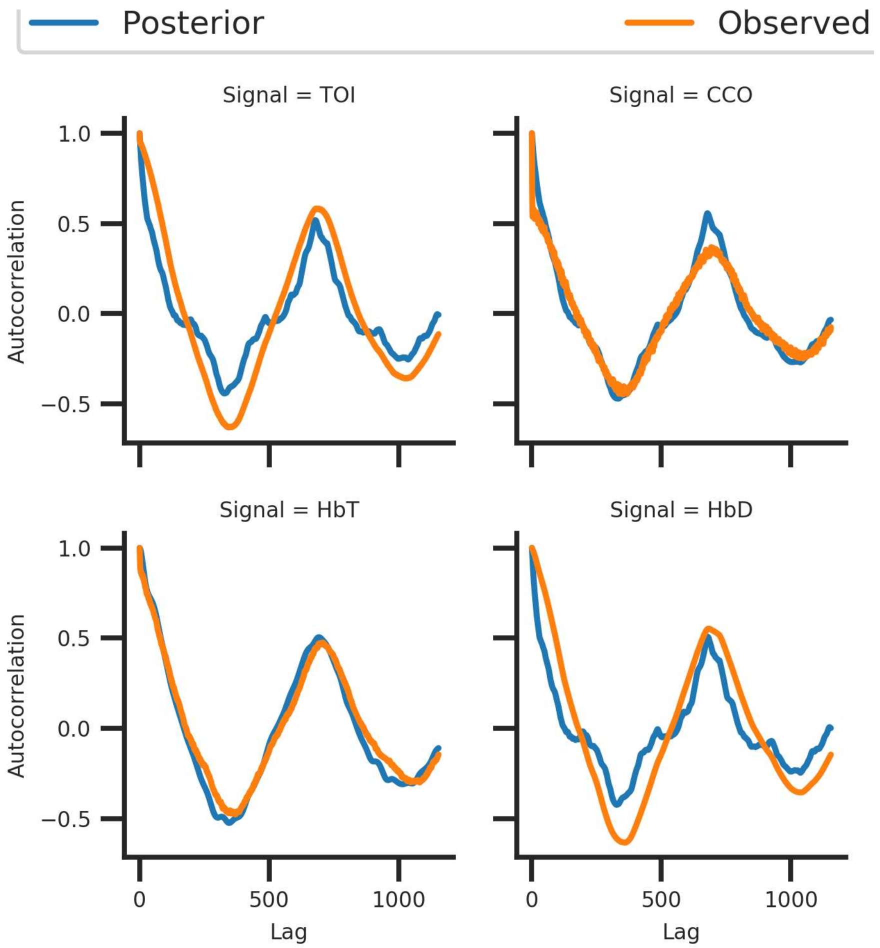

Autocorrelation of posterior predictive and the observed data. The graph shows a comparison of the autocorrelation value between observed and posterior predictive time series as a function of lag. We see significant overlap between the two suggesting an extremely good fit.
