# Supplementary material for: A Bayesian framework for the analysis of systems biology models of the brain
Source: PLoS Comput Biol. 2019 Apr 26;15(4):e1006631. doi: 10.1371/journal.pcbi.1006631 (PMC6505968; doi:10.1371/journal.pcbi.1006631)
Supplement: S14 Fig — (PDF) [file pcbi.1006631.s017.pdf]

S14 Fig Comparison of marginal posterior and prior distributions for the experimental data.

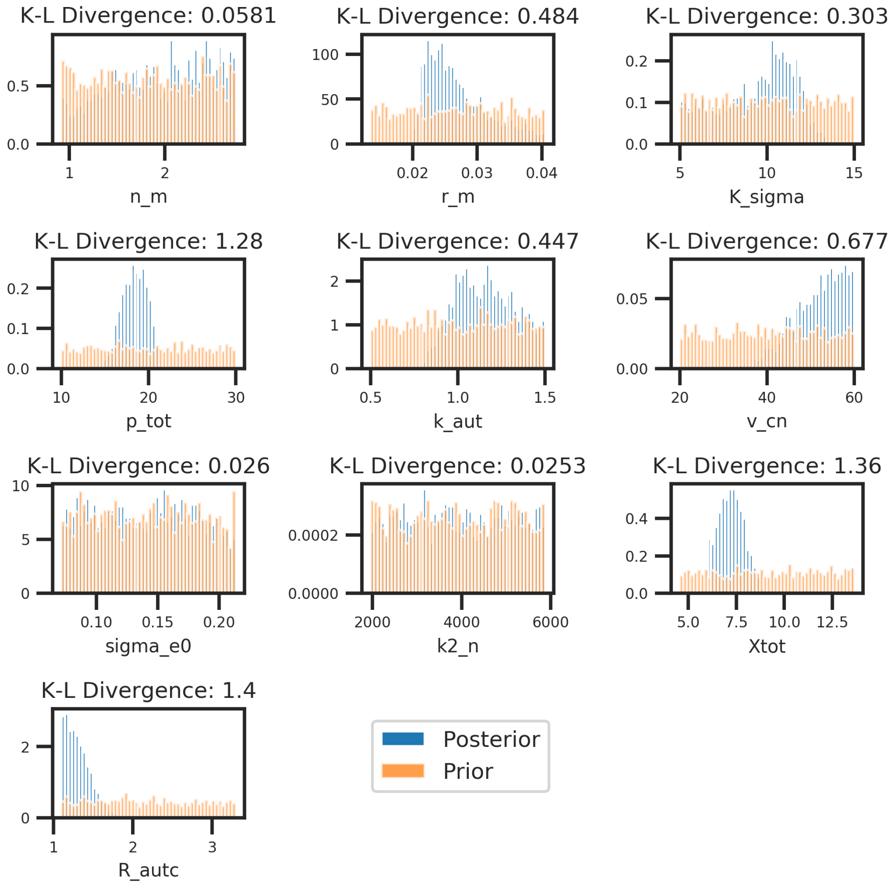

Comparison of marginal posterior and prior distributions for each parameter. A clearer comparison is made here between the marginal prior and posterior distributions for eacg fitted parameter. The Kullback–Leibler divergence is shown for each.
